# Systematic adolescent-centered supportive care program is associated with improved psychological status, treatment adherence, and social adaptation in adolescents with thyroid cancer undergoing radioiodine-131 therapy: a retrospective before-after cohort study with historical controls

**DOI:** 10.3389/fendo.2026.1901279

**Published:** 2026-09-10

**Authors:** Guoxiu Lu, Qi Peng, Jingjing Liang, Qiwen Zhong, Guoxu Zhang

**Affiliations:** 1 Department of Nuclear Medicine, General Hospital of Northern Theatre Command, Shenyang, China; 2 Training Base for Graduate, General Hospital of Northern Theater Command, China Medical University, Shenyang, China; 3 Training Base for Graduate, General Hospital of Northern Theater Command, DaLian Medical University, Shenyang, China

**Keywords:** adolescent and young adult, differentiated thyroid cancer, narrative medicine, radioiodine−131 therapy, social adaptation

## Abstract

**Background:**

Adolescents and young adults (AYAs) with differentiated thyroid cancer (DTC) undergoing radioiodine-131 (^131^I) therapy endure unique developmental and psychosocial challenges, yet evidence for adolescent-tailored supportive interventions remains scarce. This retrospective before-after cohort program is associated with improvements in psychological distress, treatment adherence, and social reintegration.

**Methods:**

We enrolled 220 eligible patients aged 13–25 years with DTC receiving^131^I therapy (2016-2024). The control group (n=85) received routine care, while the observation group (n=135) received a multi-component supportive care program. It included narrative medicine interviews, radiation education, fertility and career counseling, social adaptation training, and sustained follow-up. Primary outcomes were anxiety (SAS) and depression (SDS) scores. Secondary outcomes covered adherence, social adaptation, fertility cognition and satisfaction. Linear mixed-effects models analyzed repeated SAS/SDS data; propensity score inverse probability weighting(IPTW), multivariable regression and Benjamini–Hochberg FDR correction were applied to adjust confounders and handle multiple comparisons.

**Results:**

In linear mixed-effects models, the observation group exhibited significantly lower 6-month SAS and SDS scores (all adjusted P < 0.001).Significant group×time interactions for both scales demonstrated sustained psychological improvements. In multivariable logistic regression analyses adjusting for potential confounders, the observation group also achieved superior treatment adherence( aOR=2.71 ,95%CI:(1.52–4.83), school/work resumption rates (aOR=2.35,95%CI:1.22−4.51), and medical satisfaction (aOR=2.01,95%CI:1.11−3.64) (allFDR−P<0.05).IPTW sensitivity analyses corroborated these findings. Exploratory subgroup analyses did not reveal consistent evidence of differential intervention effects across most subgroups.

**Conclusion:**

A standardized adolescent-centered supportive care program was associated with persistent anxiety and depression relief, improved adherence and social reintegration among AYAs with DTC receiving ^131^I therapy. While these real-world findings support program feasibility, prospective multicenter randomized trials are required to confirm causal efficacy.

## 1 Introduction

Thyroid cancer is one of the most rapidly increasing malignancies worldwide (1), with a marked rise in incidence among adolescents and young adults (AYAs) in recent decades. Differentiated thyroid cancer (DTC) accounts for over 90% of all thyroid malignancies (2), and radioiodine-131 (¹³¹I) therapy is a standard adjuvant treatment following thyroidectomy for high-risk DTC (3, 4). Although ¹³¹I therapy achieves excellent oncological efficacy with mild physical side effects, mandatory isolated hospitalization and long-term radiation exposure concerns create unique psychological and developmental pressures for adolescent patients.

Epidemiological global data demonstrated thyroid cancer has become the second most common malignant tumor in female AYAs and the top diagnosed cancer in male AYAs by 2022 (5), with annual incidence growth rates reaching 3% (20–39 years old) and 4% (15–19 years old) (6). Adolescence is a critical developmental period characterized by identity formation, social integration, and future academic/career planning. Adolescent DTC survivors face unique stressors including radiation stigma, fear of social exclusion, worries about school interruption, occupational restrictions, and long-term fertility impairment risks (7–9). Previous studies have reported high rates of anxiety and depression in this population, with psychological distress often exceeding that observed in adult patients (10, 11). A cross-sectional surveys reported anxiety prevalence up to 62.3% in adolescent thyroid patients, substantially exceeding psychological burden in adult thyroid cancer populations (12). Despite growing recognition of these challenges, current routine clinical care remains disease-centered, lacking holistic, developmentally tailored psychosocial support targeting adolescent-specific unmet needs (13, 14).

Conventional adolescent-centered supportive nursing and psychological counseling have proven beneficial for adult cancer survivors’ mental health, but comprehensive, adolescent-oriented systematic supportive intervention packages integrating narrative medicine, occupational guidance, and fertility consultation remain understudied in the context of ^131^I therapy (15). And evidence regarding systematic supportive interventions specifically for adolescents undergoing ¹³¹I therapy remains scarce (16). After oral administration of radioactive iodine-131,the drug is selectively taken up by residual thyroid tissue and metastatic lesions, destroying diseased tissue through the emission of β-rays. This treatment is simple, effective, and has mild side effects (17, 18), and has become a standard therapeutic approach after radical thyroidectomy as recommended by guidelines (19). Patients need physical isolation (usually 1–2 weeks) during treatment and maintain an appropriate distance from others for a period after treatment (20). Existing research focuses mainly on psychological screening or simple health education, lacking integrated models that address the full spectrum of adolescent-specific needs, including career development, fertility preservation, and social reintegration (21).

To address this gap, the present study developed and implemented a systematic adolescent-centered supportive care program for adolescents with DTC receiving ¹³¹I therapy. The primary objective was to evaluate the associations between this program and psychological status, treatment adherence, social adaptation, and long-term quality of life. Secondary objectives included exploring subgroups that may benefit most from the intervention in an exploratory manner. Findings aim to provide preliminary real-world evidence for incorporating structured adolescent supportive care into routine nuclear medicine clinical workflows and inform the design of future prospective interventional trials.

## 2 Subjects and methods

### 2.1 Study design

This was a single-center retrospective before-after cohort study utilizing historical routine-care patients as the control group. All clinical data were retrospectively extracted from standardized hospital electronic medical record systems between January 2016 and December 2024. All personal identifying information was permanently anonymized before analysis to fully protect patient privacy. The systematic adolescent-centered supportive care program was formally launched as a hospital-wide standardized clinical policy in July 2020, rather than a research-specific prospective intervention. The studies involving human participants were reviewed and approved by the Ethics Committee of the General Hospital of Northern Theater Command, Shenyang, China (Approval No. YL2021-07) (**Supplementary Material 1**: full English translation of approval document), waiving the requirement for written informed consent from both adolescent patients and their legal guardians for retrospective anonymized data extraction, as the study posed no additional risks to participants and all data were de-identified.

Original clinical admission routine treatment consent was obtained from all patients/guardians at hospital discharge, which was independent of this retrospective observational analysis project. Baseline demographic characteristics and clinical indicators of the two groups were statistically compared prior to analysis; the standardized mean differences (SMDs) for all variables were < 0.1, indicating good balance on measured covariates, though unmeasured temporal secular confounding factors cannot be fully eliminated due to the non-randomized historical-control design.

### 2.2 Study subjects

Inclusion criteria: (1) age range 13 to 25 years at time of ^131^I therapy; (2) histopathologically confirmation of papillary or follicular differentiated thyroid carcinoma; (3) met clinical indications for radioactive iodine-131 therapy; (4) no severe irreversible dysfunction of cardiac, hepatic, renal, or other vital organs; (5) complete baseline clinical, laboratory, and scale assessment data; and willingness to complete standardized 1-, 3-, 6-, 12 and 24-month follow-up assessments.

Exclusion criteria: (1) pregnant or lactating female patients; (2) concurrent primary malignant tumors of any organ system; (3) severe mental illnesses affecting cooperation with treatment and study procedures; (4) prior history of cervical radiotherapy; (5) incomplete baseline clinical data or inability to complete follow-up at any scheduled assessment timepoint.

**Figure 1:**
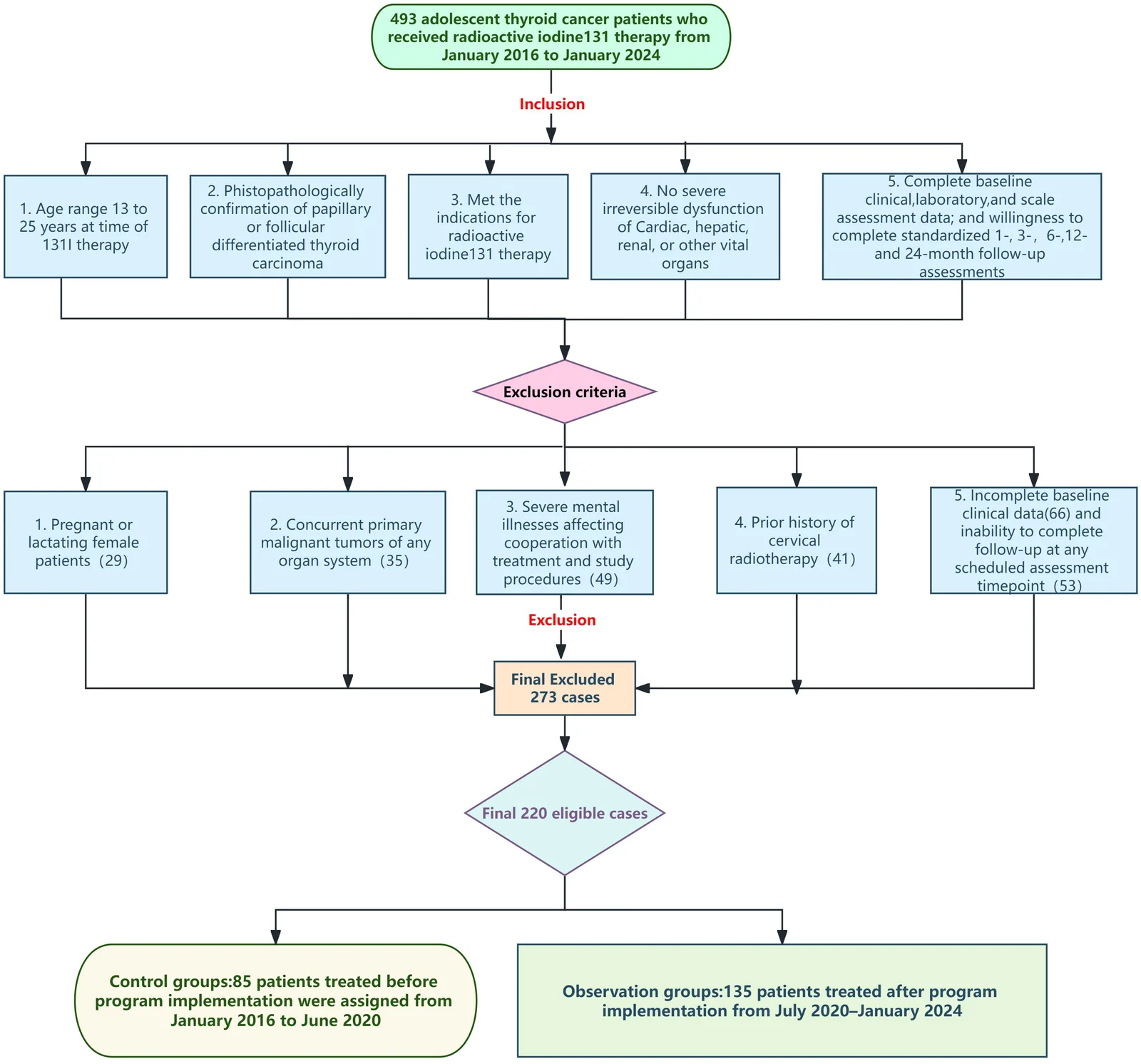
Participant enrollment flowchart. A total of 493 adolescent thyroid cancer patients who received radioactive iodine-131 therapy between January 2016 and December 2024 were initially screened. After applying inclusion and exclusion criteria, 220 eligible patients were finally included. Among the 220 included, 85 patients treated before program implementation were assigned to the control group, and 135 patients treated after program implementation were included in the observation group.

A total of 493 adolescent patients who received ¹³¹I therapy between January 2016 and December 2024 were initially screened. All subjects were strictly screened according to the predefined inclusion and exclusion criteria. The final analytic sample included 220 participants who completed all assessments with no missing data for the variables of interest (a complete-case cohort). Participants were grouped by clinical care policy timeline: Control group (n=85, January 2016–June 2020, routine care without systematic supportive program); Observation group (n=135, July 2020–December 2024, full systematic adolescent-centered supportive care package). The complete participant screening flowchart is presented in **Figure 1**.

### 2.3 Grouping and interventions

#### 2.3.1 Control group (n=85, routine standard care)

All patients in the control group received the routine radioactive iodine-131 therapy process. Pre-treatment evaluation included thyroid function, blood routine, liver and kidney biochemistry, electrocardiogram. Clinicians briefly introduced ¹³¹I therapeutic principle, expected side effects, and basic isolation precautions via printed standard education leaflets. Leaflets covered mandatory isolation duration, household distancing rules, and 6–12 month contraception recommendations. Scheduled follow-up timepoints (1/3/6/12 months) were verbally notified. Post-treatment follow-up relied on routine outpatient review or brief telephone check-ins. No narrative psychological interviews, targeted radiation fear counseling, career/fertility guidance, or family intervention services were provided.

#### 2.3.2 Observation group (n=135, systematic adolescent-centered supportive care program)

**Figure 2:**
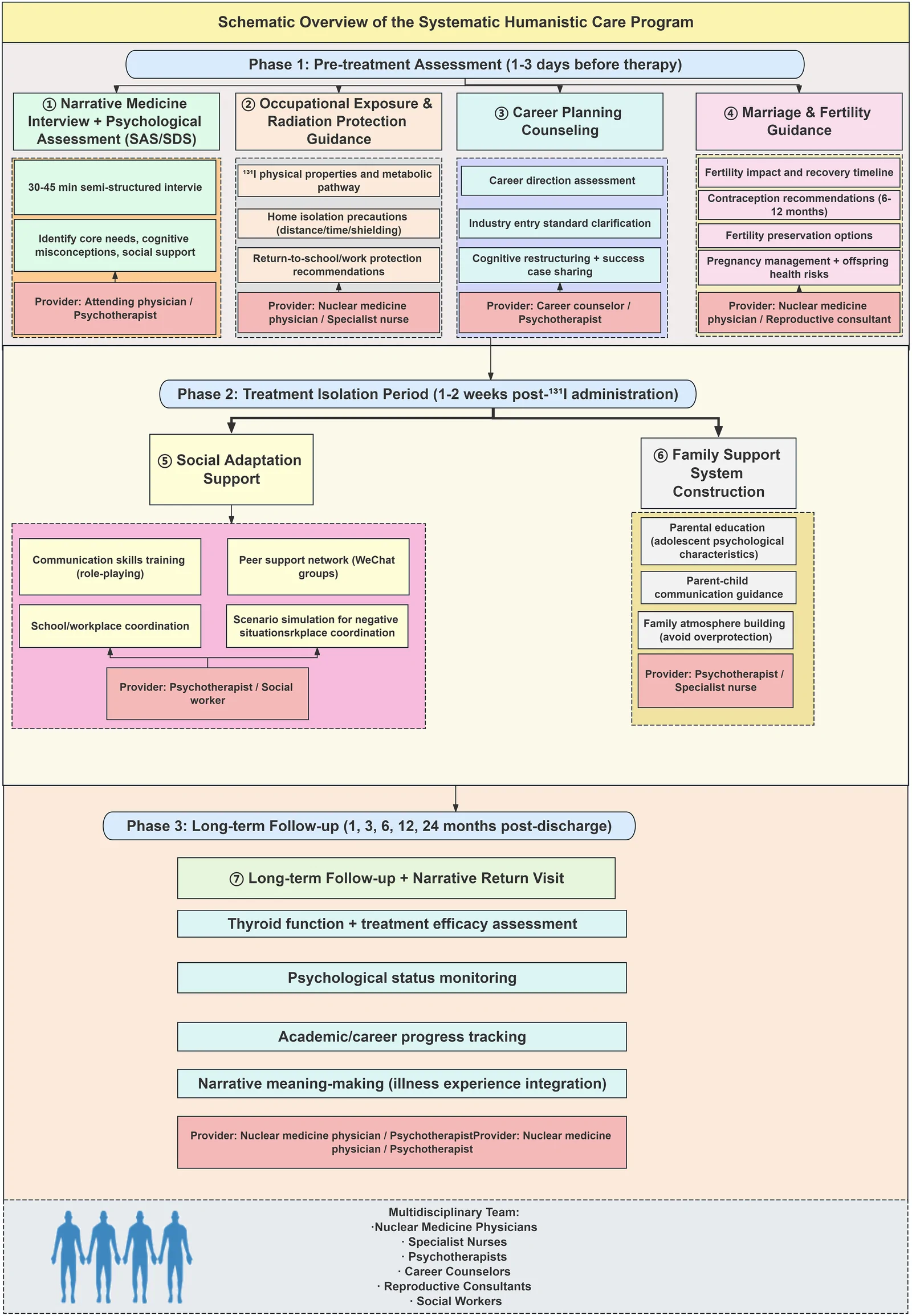
Flowchart of the standardized multidisciplinary supportive care program during radioiodine-131 treatment. This flowchart presents the multidisciplinary care framework consisting of pre-treatment assessment, in-hospital isolation intervention, and long-term post-discharge follow-up, integrating narrative medicine, psychological intervention, radiation protection guidance, and fertility counseling for adolescent thyroid cancer patients.

All patients in the observation group received systematic adolescent-centered supportive care on the basis of routine treatment. All routine thyroid and radioiodine management was identical to controls. Seven standardized multi-module supportive interventions were delivered by a fixed multidisciplinary team (nuclear medicine physicians, specialized nurses, licensed psychological counselors, career consultants, reproductive specialists). The full intervention schematic diagram of the intervention program is provided in **Figure 2**.

1. Narrative Medicine Interviews and Baseline Psychological Assessment

Before treatment decision-making, a 30–45 minute narrative interview was conducted by the attending physician or trained psychological counselor using a semi-structured format focusing on core questions (**Supplementary Material 2**). During the interview, the interviewer used active listening, empathetic responses,and open-ended questions to encourage patients to express themselves fully. After the interview, the interviewer identified the patients’ core demands, cognitive misunderstandings, psychological status,and social support based on their narratives. Meanwhile, baseline assessment was performed using the SAS (22) and SDS (23).

2. Occupational Exposure and Protection Guidance

To address the common fear of “radiation”, scientific radiation protection guidance was provided. It included: (1) physical properties and metabolism of radioactive iodine-131 (half-life 8.02 days, metabolic pathway, radiation type); attenuation of internal radioactivity over time (visual “radiation level over time chart”); specific protection measures during home isolation (distance, time, shielding); protection suggestions for returning to school/work; and employment restrictions for special occupations with reference to authoritative policy documents (**Supplementary Material 3**).

3. Individual Career Planning Counseling

Age-adapted career planning counseling aimed to help patients understand the impact of the disease on career choices and re-plan their life paths, including:career direction assessment; career limitation clarification; cognitive restructuring to address “illness-induced self-limitation”; peer survivor success story sharing from former patients and academic resumption plans for student patients (**Supplementary Material 4**).

4. Marriage and Fertility Guidance

Evidence-based reproductive health education referencing ATA (24) and Chinese CSNM thyroid guidelines (19), including contraception advice (25); Fertility preservation options (26); pregnancy management (27); offspring health risks (28, 29); lactation guidance and psychological support for fertility fears (30).

5. Social Adaptation Training

Practical guidance was provided for social dilemmas after returning to school/work: (1) Communication skill training (31): Role-playing to help patients master how to explain their condition to classmates/colleagues, including what to say, how to say, and when to say. (2) School/work coordination (32): Communicating with counselors, head teachers, or HR departments with patients’ consent to explain the real situation and strive for reasonable arrangements (leave during isolation, normal treatment after return, necessary protection without special treatment). (3) Peer support network establishment (33): Organizing exchanges among patients with similar experiences to form a mutual aid network (WeChat groups, offline gatherings when protection permits). (4) Scenario simulation (34, 35) training for coping with negative situations.

6. Family Support System Construction

Family support is the most important social resource for adolescent patients. Synchronous guidance was provided to family members (36–39), including: (1) Parental education on adolescent psychological characteristics;(2) Communication guidance: Instructing parents on how to discuss the condition, express care without causing anxiety, and listen to patients’ true thoughts.(3)Family atmosphere building to avoid over-protection or over-anxiety, and create a supportive rather than oppressive atmosphere. (4) Family relationship coordination: Assisting family communication for patients with tense family relationships due to illness to alleviate conflicts.

7. Long-term Follow-up and Narrative Return Visits.

The observation group received more frequent follow-up at 1 month, 3 months, 6 months, 1 year, and 2 years after treatment, covering thyroid function, treatment effect, psychological status, academic/career progress, fertility plan, and social adaptation. Narrative return visits integrated narrative interviews into follow-up to help patients integrate the disease experience into their life stories (**Supplementary Material 5**).

### 2.4 Outcome measures and assessment tools

#### 2.4.1 Primary outcomes (SAS and SDS)

Assessed using SAS and SDS: (1) SAS: 20 items,4-level scoring; ≥50 indicates anxiety (Cronbach’s α=0.86 in Chinese adolescents). (2) SDS: 20 items,4-level scoring; ≥53 indicates depression (Cronbach’s α=0.85 in Chinese adolescent). Assessed at baseline, 1 month, 6 months, and 12 months after treatment (**Supplementary Material 6**).

#### 2.4.2 Secondary outcomes

1. Treatment-related Knowledge Mastery

Self-designed questionnaire (15 items) covering treatment principle, radiation protection, isolation requirements, side effect identification, follow-up schedule, occupational impact, and fertility impact. Full score 100; ≥85=excellent,60–84=qualified,<60=unqualified (Cronbach’s α=0.89, CVI = 0.92). The questionnaire was developed through literature review and expert panel review (5 experts including pediatric endocrinologists, nuclear medicine physicians, and psychologists), then pilot-tested in 30 adolescent thyroid cancer patients prior to the study. Assessed 1 month after treatment (**Supplementary Material 7**).

2. Treatment Compliance, Social Adaptation, Career Planning Clarity

Treatment compliance was defined as: (a) timely intake of the prescribed radioiodine-131 dose, (b) compliance with isolation requirements during the post-treatment period, and (c) attendance at scheduled follow-up visits. All “yes” = good compliance; one or more “no” = poor compliance. Assessed by attending physicians based on objective medical record documentation.

3. Social Adaptation

Social adaptation was assessed using a self-designed questionnaire covering return-to-school/return-to-work status, social participation (5-level), and relationship satisfaction (5-level). The questionnaire was developed through literature review and expert validation (CVI = 0.90), pilot-tested in 30 patients.

4. Career Planning Clarity

Career planning clarity was assessed using a single item: “I have a clear plan for future career development” (5-level scoring). Single-item measures were interpreted with appropriate caution.

5. Fertility cognition and confidence

Fertility cognition and confidence were assessed using a self-designed questionnaire including fertility knowledge mastery (5 items, ≥80=excellent) and fertility confidence (5-level scoring). Developed through literature review and expert validation (CVI = 0.88).

6. Overall satisfaction

Overall satisfaction: 5-level composite scale covering doctor-patient communication, psychological support, information provision; satisfaction rate = proportion selecting “satisfied/very satisfied”. All secondary outcomes measured at 6 months post-ablation. All self-designed scales underwent expert panel review and pre-test on 30 adolescent thyroid patients before formal data collection; full questionnaire texts attached to **Supplementary Materials** with in-text citations (**Supplementary Material 8**).

### 2.5 Statistical methods

Post-hoc power analysis via G*Power 3.1 (α=0.05,d=0.5,n1 = 85,n2 = 135) showed statistical power (1−β)=0.92,sufficient to detect moderate effect sizes. SPSS 26.0 and R4.2.2 were used for analysis. Descriptive statistics were presented as mean ± standard deviation for continuous data and number (percentage) for categorical data. For repeated measures of SAS and SDS, we fitted linear mixed-effects models with group (intervention vs. control), time (categorical: 1, 6, and 12 months), and their interaction as fixed effects, and a random intercept for each participant to account for within-subject correlation. Baseline scores of the respective outcome were included as a covariate to adjust for potential regression to the mean and initial differences. Models were estimated using restricted maximum likelihood (REML) with an unstructured covariance matrix for the repeated measurements, which was selected based on the lowest Akaike information criterion (AIC) and Bayesian information criterion (BIC) among candidate structures (compound symmetry, first-order autoregressive, and unstructured). Degrees of freedom were computed using the Satterthwaite approximation. The primary test of intervention effect was the group-by-time interaction. Estimated marginal means, adjusted mean differences between groups at each time point with 95% confidence intervals, and model fit statistics (-2 log likelihood, AIC, BIC) are reported in the main text and in **Supplementary Table 1**. The variance components of the random effects are also provided in **Supplementary Table 1**.

For secondary outcomes, multivariable logistic regression adjusting for potential confounders (age, gender, TNM stage, RAI dose, time since diagnosis, education, residence, occupation, baseline SAS/SDS) were adjusted analyses.

To account for potential confounding due to the slight baseline imbalance in occupation, all multivariable models were adjusted for occupation along with other covariates. Furthermore, we employed propensity score inverse probability of treatment weighting (IPTW) (40) as a sensitivity analysis. The IPTW model included all covariates; post-weighting balance was assessed, with SMD < 0.1 indicating excellent balance (41). This approach ensures that our findings are robust to observed confounders. After weighting, the effective sample size and distribution of weights were examined, and we observed no extreme weights (all weights < 10 times the mean). The weighted analyses produced effect estimates and 95% CIs.

Multiple comparisons as below: SAS and SDS were designated as primary outcomes. For secondary outcomes, Benjamini-Hochberg false discovery rate (FDR) correction was applied. FDR-adjusted P values are reported. Subgroup analyses as below: Formal interaction tests were performed by including group-by-subgroup interaction terms in regression models. Interaction P values and effect estimates with 95% confidence intervals are reported. Subgroup findings are presented as exploratory. *P* < 0.05 (two-tailed) was considered statistically significant. For secondary outcomes, FDR-adjusted *P* < 0.05 was required.

## 3 Results

### 3.1 Baseline characteristics

The control group (n=85) included 32 males (37.6%) and 53 females (62.4%),mean age 19.48 ± 3.60 years. The observation group (n=135) included 54 males (40.0%) and 81 females (60.0%),mean age 18.97 ± 3.87 years. Baseline characteristics of the two groups are summarized in **Table 1**. The standardized mean differences (SMDs) for most covariates were below 0.2, indicating generally good balance. However, the SMD for occupation was 0.29, which exceeds the commonly recommended threshold of 0.20, suggesting a slight imbalance in the distribution of student versus non-student status between the historical control and observation groups. To address this and other potential baseline differences, all subsequent analyses were adjusted for the full set of covariates listed in **Table 1** using multivariable regression. Furthermore, we applied propensity score inverse probability of treatment weighting (IPTW) as a sensitivity analysis; after weighting, all post-weighting SMDs were < 0.1,confirming that the weighting procedure successfully balanced the groups on measured covariates. Therefore, while a minor baseline imbalance existed, the primary and sensitivity analyses both supported the robustness of our findings. **Table 1** presents baseline characteristics.

**Table 1:** Baseline characteristics of the two groups.

Characteristics	Control group (n=85)	Observation group (n=135)	Test statistic	P value	Unweighted SMD	Weighted SMD
Age (years)	19.48 ± 3.60	18.97 ± 3.87	t=0.98	0.326	0.14	0.03
Gender, n (%)			χ²=0.12	0.722	0.05	0.02
Male	32 (37.6)	54 (40.0)				
Female	53 (62.4)	81 (60.0)				
Occupation, n (%)			χ²=4.21	0.240	0.29	0.06
Student	35 (41.2)	75 (55.6)				
Military	19 (22.4)	23 (17.0)				
Staff	14 (16.5)	18 (13.3)				
Others	17 (20.0)	19 (14.1)				
Pathological type, n (%)			χ²=0.76	0.383	0.12	0.04
Papillary	72 (84.7)	108 (80.0)				
Follicular	13 (15.3)	27 (20.0)				
TNM stage (42), n (%)			χ²=1.24	0.743	0.15	0.05
I	38 (44.7)	62 (45.9)				
II	25 (29.4	43 (31.9)				
III	14 (16.5)	19 (14.1)				
IV	8 (9.4)	11 (8.1)				
RAI dose (mCi)	102.5 ± 28.3	98.7 ± 30.1	t=0.94	0.348	0.13	0.04
Time since diagnosis (months)	3.8 ± 1.6	4.1 ± 1.8	t=1.24	0.216	0.18	0.05
Education, n (%)			χ²=2.02	0.364	0.19	0.06
Middle school or below	8 (9.4)	9 (6.7)				
High school	25 (29.4)	31 (22.9)				
College or above	52 (61.2)	95 (70.4)				
Residence, n (%)			χ²=0.36	0.547	0.08	0.03
Urban	52(61.2)	88(65.2)				
Rural	33(38.8)	47(34.8)				
TSH (mIU/L)	3.57 ± 0.81	3.60 ± 0.84	t=0.24	0.804	0.04	0.02
SAS score(n)	47.69 ± 6.01	47.47 ± 5.93	t=-0.27	0.783	0.04	0.03
SDS score(n)	40.46 ± 4.64	40.30 ± 4.71	t=-0.25	0.802	0.03	0.02
Social support	34.59 ± 2.14	35.02 ± 2.22	t=1.38	0.166	0.20	0.07

SAS, Self-Rating Anxiety Scale; SDS, Self-Rating Depression Scale; RAI, radioiodine; TNM, tumor-node-metastasis. Continuous data presented as mean ± standard deviation; categorical data as n (%). Independent-samples t-test andχ²test were used. SMD = standardized mean difference. SMD < 0.1 is generally considered to indicate excellent balance between groups. IPTW was performed using a logistic regression model that included all covariates listed above. The effective sample size after weighting was 198.5 (control: 72.3; intervention: 126.2), and no extreme weights were observed (all weights < 10 times the mean). The post-weighting SMDs confirmed that the weighting procedure successfully balanced all measured covariates between the two groups.

### 3.2 Primary outcomes: longitudinal SAS and SDS scores

#### 3.2.1 SAS scores

**Figure 3:**
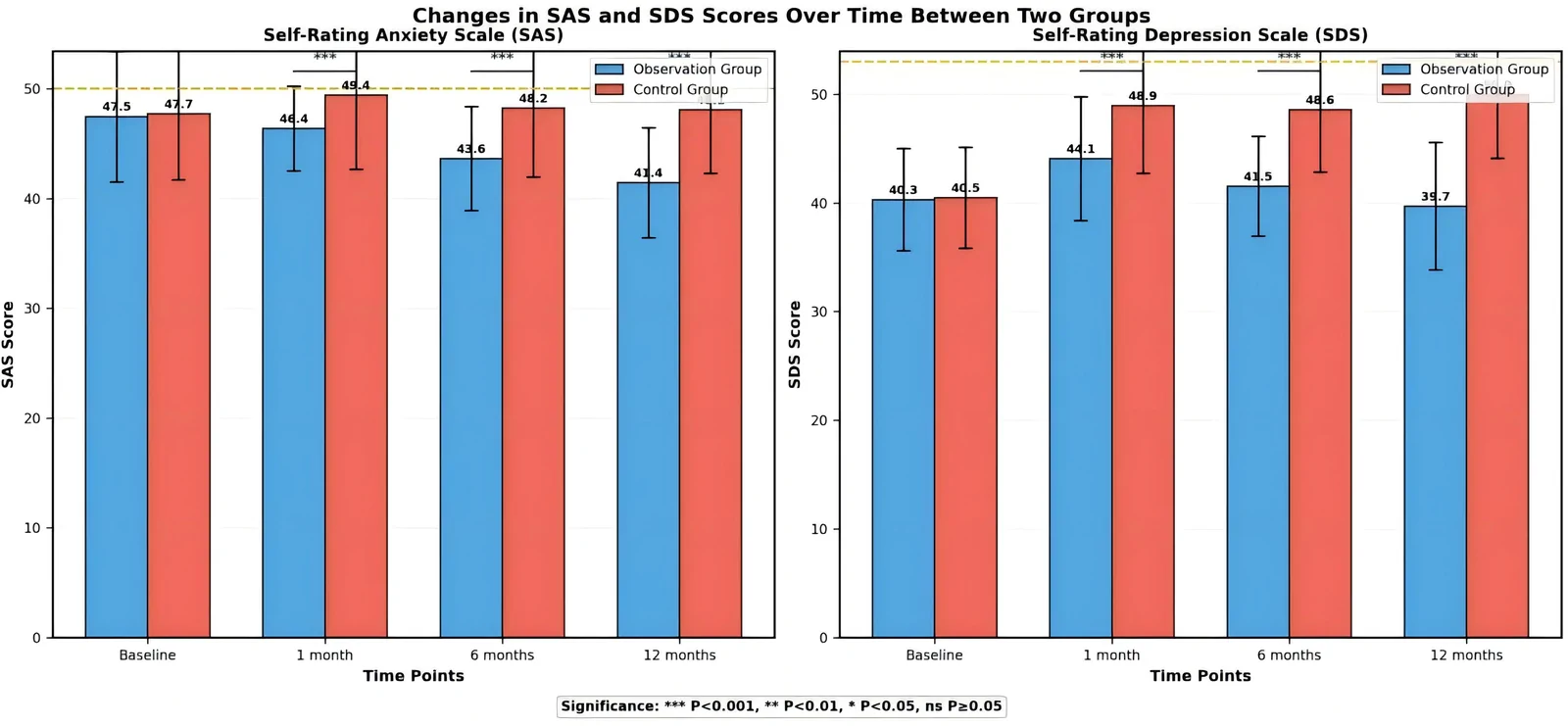
Comparisons of self-rating anxiety scale (SAS) and self-rating depression scale (SDS) scores between the observation group and control group at different time points. Data are presented as mean ± standard deviation. The x-axis represents the four assessment time points: Baseline, 1 month, 6 months, and 12 months post-intervention. The y-axis represents scale scores. Orange dashed lines indicate normal cutoff values (SAS < 50; SDS < 53). ***P < 0.001; ns, non-significant (P ≥ 0.05). Error bars represent standard deviations. Linear mixed-effects models with group×time interaction were used for primary analysis; cross-sectional t-tests shown for visualization.

Linear mixed-effects analysis revealed a significant group×time interaction for SAS scores( F=18.42,P<0.001), indicating that the change in anxiety over time differed significantly between groups. At baseline, SAS scores were comparable (control: 47.69 ± 6.01; observation: 47.47 ± 5.93; P = 0.783). At 1 month post-treatment, the observation group had significantly lower SAS scores (46.37 ± 3.86 vs. 49.42 ± 6.77; adjusted P < 0.001; Cohen’s d = 0.59, 95% CI: 0.31-0.87). At 6 months post-treatment, the observation group continued to show lower scores (43.64 ± 5.70 vs. 48.24 ± 6.28; adjusted P < 0.001; d = 0.78, 95% CI: 0.50–1.06). At 12 months, the difference was sustained (41.42 ± 4.56 vs. 48.82 ± 5.87; adjusted P < 0.001; d = 1.45, 95% CI: 1.15–1.75).The adjusted mean difference (intervention minus control) at 6 months was-4.60 (95% CI: -6.12 to-3.08, P<0.001), and at 12 months it was-7.40 (95% CI:-8.92 to -5.88, P<0.001). The proportion of patients with normal SAS scores (<50) at 6 months was significantly higher in the observation group (94.8%vs.71.8%;χ²=21.03, P < 0.001, Cramer’s V = 0.31) (**Figure 3**).

#### 3.2.2 SDS scores

Linear mixed-effects analysis revealed a significant group×time interaction for SDS scores( F=22.56,P<0.001). At baseline, scores were comparable (control: 40.46 ± 4.64; observation: 40.30 ± 4.71; P = 0.802). At 1 month post-treatment, the observation group had significantly lower SDS scores (44.08 ± 5.69 vs. 48.94 ± 6.23; adjusted P < 0.001; d = 0.82, 95% CI: 0.54–1.11). At 6 months, the difference was more pronounced (41.54 ± 4.60 vs. 48.56 ± 5.74; adjusted P < 0.001; d = 1.39, 95% CI: 1.08–1.69). At 12 months, the benefit was maintained (40.92 ± 4.38 vs. 48.11 ± 5.42; adjusted P < 0.001; d = 1.45, 95% CI: 1.15–1.75).The adjusted mean difference (intervention minus control) at 6 months was-4.23 (95% CI: -5.94 to-3.17, P<0.001), and at 12 months it was-4.25 (95% CI:-6.13 to -2.95, P<0.001). The proportion with normal SDS scores (<53) at 6 months was significantly higher in the observation group (96.3% vs. 85.9%; χ²=6.54, P = 0.011, Cramer’s V = 0.17) (**Figure 3**).

### 3.3 Secondary outcomes

#### 3.3.1 Treatment-related knowledge mastery

The observation group achieved an Excellent+Good rate of 77.04% (104/135), significantly higher than the control group (63.53%, 54/85;χ²=4.702, FDR-adjusted P = 0.030). The observation group had higher correct rates in radiation protection, isolation precautions, diet management, follow-up schedule, occupational impact, and fertility advice (all FDR-adjusted P < 0.01) (**Table 2**).

#### 3.3.2 Treatment compliance

The good treatment compliance rate in the observation group was 78.6% (106/135), significantly higher than 55.3% (47/85) in the control group (χ²= 13.283, FDR-adjusted P < 0.001). Only 5 patients (3.7%) in the observation group discontinued treatment due to psychological concerns, compared with 8 patients (9.4%) in the control group (**Table 2**).

#### 3.3.3 Social adaptation

The return-to-school/return-to-work rate in the observation group reached 90.4% (122/135),significantly higher than 78.8% (67/85) in the control group (χ² = 5.744, FDR-adjusted *P* = 0.003). The social participation score was 4.28 ± 0.31 in the observation group versus 3.72 ± 0.29 in the control group(t=13.577, FDR-adjusted *P* < 0.001). The relationship satisfaction score was 4.15 ± 0.27versus 3.58 ± 0.32 (t=13.646, FDR-adjusted *P* < 0.001). The normal collective activity participation rate was 84.4% in the observation group versus 65.9% in the control group (χ²=10.232, FDR-adjusted *P* < 0.001) (**Table 2**).

#### 3.3.4 Career planning clarity

The career planning clarity score was 4.12 ± 0.36 in the observation group versus 3.05 ± 0.41 in the control group (t=19.742, FDR-adjusted *P* < 0.001). The career confidence rate was 72.6% (98/135) in the observation group versus 52.9% (45/85) in the control group (χ²=8.854 FDR-adjusted *P* = 0.001) (**Table 2**).

#### 3.3.5 Fertility cognition and confidence

The fertility confidence rate was 76.3% (103/135) in the observation group versus75.3% (64/85) in the control group (χ²=0.029, FDR-adjusted *P* = 0.865). In the sex-stratified analysis, the intervention effect on fertility confidence was not statistically significant in female patients ( 77.8% vs. 83.0%, P=0.460) or male patients (74.1% vs. 62.5%, P = 0.251). The formal interaction test for sex did not reach statistical significance(interaction P = 0.34), indication no strong evidence of differential intervention effects between male and female patients. However, in the exploratory age-stratified analysis, a significant improvement in fertility confidence was observed in the 13–8 years subgroup (82.8% vs. 53.1%, FDR-P=0.008), with a significant age-by-group interaction( P=0.031), suggesting that younger patients may derive greater benefit in this domain (**Table 2**).

#### 3.3.6 Overall satisfaction

The overall satisfaction rate was 72.6% (98/135) in the observation group, significantly higher than 58.8% (50/85) in the control group (χ² = 4.492, *P* = 0.034).Patients in the observation group reported higher satisfaction with doctor-patient communication, psychological support, information provision, and comprehensive medical experience (**Table 2**).

**Table 2:** Comparisons of social adaptation, treatment compliance, career and fertility outcomes between two groups.

Variables	Observation group (n=135)	Control group (n=85)	Test statistic	FDR- adjust P-value
Social participation score, Mean ± SD	4.28 ± 0.31	3.72 ± 0.29	t=13.577	<0.001
Relationship satisfaction score, Mean ± SD	4.15 ± 0.27	3.58 ± 0.32	t=13.646	<0.001
Career planning clarity score, Mean ± SD	4.12 ± 0.36	3.05 ± 0.41	t=19.742	<0.001
Good treatment compliance, n (%)	106 (78.5%)	47 (55.3%)	χ²=13.283	<0.001
Excellent+Good rate, n (%)	104(77.0%)	54(63.5%)	χ²=4.702	0.045
Return-to-school/return-to-work,n (%)	122 (90.4%)	67 (78.8%)	χ²=5.744	0.020
Normal collective activity participation, n (%)	114 (84.4%)	56 (65.9%)	χ²=10.232	0.004
Career confidence, n (%)	98 (72.6%)	45 (52.9%)	χ²=8.854	0.006
Fertility confidence, n (%)	103 (76.3%)	64 (75.3%)	χ²=0.029	0.865
Overall satisfaction, n (%)	98 (72.6%)	50 (58.8%)	χ²=4.492	0.040

Continuous data presented as mean ± standard deviation and compared using independent-samples t-test. Categorical data presented as n (%) and compared using χ² test. FDR, false discovery rate.

### 3.4 Propensity score IPTW sensitivity analysis

The IPTW procedure was performed using a logistic regression model that included all covariates listed in **Table 1**. The model showed a C-statistic of 0.61 (95% CI: 0.53–0.69),indicating modest predictive discrimination. After weighting, the effective sample size was 198.5 (control: 72.3; intervention: 126.2). The stabilized weights ranged from 0.52 to 2.38 mean=1.00, with no extreme values (all<10 times the mean). Balance diagnostics showed that all post-weighting SMDs were <0.1, indicating excellent covariate balance. The weighted logistic regression yielded adjusted odds ratios (95% CI) for treatment compliance, return-to-school/work, and overall satisfaction of 2.71 (1.52–4.83), 2.35 (1.22–4.51), and 2.01 (1.11–3.64), respectively (all P<0.05). These estimates are consistent with the primary multivariable-adjusted results, confirming that the slight baseline imbalance in occupation did not materially affect our conclusions.

### 3.5 Subgroup analyses

To explore potential heterogeneity in intervention effects, we performed subgroup analyses stratified by age, gender, social support level, and metastasis status. Baseline characteristics within each subgroup were balanced between groups (**Table 3**). Formal interaction tests were conducted by including group-by-subgroup interaction terms in regression models; all subgroup P values and interaction P values were adjusted for multiple comparisons using the FDR method (**Table 4**).

**Table 3:** Baseline characteristics of each subgroup.

Characteristics	Category	Control(n=85)	Observation(n=135)	Test statistic	P-value	SMD
Age group	13–18 years	32(37.6%)	58(43.0%)	χ²=0.410	0.522	0.108
	19–25 years	53(62.4%)	77(57.0%)			0.108
	Cramér’s V	–	–	–	–	0.043
Gender	Male	32(37.6%)	54(40.0%)	χ²=0.118	0.731	0.048
	Cramér’s V	–	–	–	–	0.023
Social support	Low	29(34.1%)	46(34.1%)	χ²=1.378	0.502	0.001
	Moderate	35(41.2%)	59(43.7%)			0.051
	High	21(24.7%)	30(22.2%)			0.059
	Cramér’s V	–	–	–	–	0.056
Metastasis status	No metastasis	42 (49.4%)	68 (50.4%)	χ²=0.867	0.833	0.019
	Cervical lymph node	25 (29.4%)	39 (28.9%)			0.012
	Lung metastasis	12 (14.1%)	18 (13.3%)			0.023
	Cervical lymph node + Lung metastasis	6 (7.1%)	10 (7.4%)			0.013
	Cramér’s V	–	–	–	–	0.036
SAS score	Mean ± SD	47.69 ± 6.01	47.47 ± 5.93	t=-0.267	0.790	0.037
SDS score	Mean ± SD	40.46 ± 4.64	40.30 ± 4.71	t=-0.247	0.805	0.034

SAS, Self-Rating Anxiety Scale; SDS, Self-Rating Depression Scale. SMD < 0.1 indicates excellent balance; SMD < 0.2 indicates acceptable balance.

**Table 4:** Outcomes of subgroup analysis after intervention.

Outcome measures	Subgroup category	Subgroup level	Control (n=85)	Observation (n=135)	Test statistic	P-value	FDR- adjusted P	Effect size (95% CI)
Return-to- school/work	Age	13–18 years	24/32 (75.0%)	54/58 (93.1%)	χ²=5.85	0.016	0.037	OR=4.50 (1.24–16.40)
		19–25 years	43/53 (81.1%)	68/77 (88.3%)	χ²=1.30	0.255	0.374	OR=1.76 (0.66–4.67)
		Interaction P-value					0.256	
	Gender	Male	26/32 (81.2%)	47/54 (87.0%)	χ²=0.52	0.469	0.566	OR=1.55 (0.47–5.10)
		Female	41/53 (77.4%)	75/81 (92.6%)	χ²=6.39	0.011	0.029	OR=3.66 (1.28–10.47)
		Interaction P-value					0.289	
	Social Support	Low	20/29 (69.0%)	41/46 (89.1%)	χ²=4.76	0.029	0.061	OR=3.69 (1.09–12.46)
		Moderate	29/35 (82.9%)	52/59 (88.1%)	χ²=0.51	0.474	0.566	OR=1.54 (0.47–5.01)
		High	18/21 (85.7%)	29/30 (96.7%)	χ²=2.05	0.152	0.257	OR=4.83 (0.47–50.09)
		Interaction P-value					0.510	
	Metastasis Status	No metastasis	34/42 (81.0%)	63/68 (92.6%)	χ²=3.41	0.065	0.123	OR=2.96 (0.90–9.77)
		Cervical lymph node	20/25 (80.0%)	34/39 (87.2%)	χ²=0.60	0.440	0.563	OR=1.70 (0.44–6.60)
		Lung metastasis	9/12 (75.0%)	15/18 (83.3%)	χ²=0.31	0.576	0.660	OR=1.67 (0.28–10.09)
		Cervical + Lung metastasis	4/6 (66.7%)	10/10 (100.0%)	χ²=3.81	0.051	0.104	OR=∞ (N/A)
		Interaction P-value					0.791	
Career planning clarity	Age	13–18 years	2.81 ± 0.39	4.25 ± 0.33	t=17.68	<0.001	<0.001	d=4.09 (3.35–4.82)
		19–25 years	3.18 ± 0.43	4.03 ± 0.38	t=11.61	<0.001	<0.001	d=2.12 (1.68–2.55)
		Interaction P-value					<0.001	
	Gender	Male	3.12 ± 0.42	4.07 ± 0.37	t=10.59	<0.001	<0.001	d=2.44 (1.87–3.01)
		Female	2.98 ± 0.40	4.15 ± 0.35	t=17.38	<0.001	<0.001	d=3.16 (2.65–3.67)
		Interaction P-value					0.050	
	Social Support	Low	2.76 ± 0.38	4.31 ± 0.32	t=18.26	<0.001	<0.001	d=4.50 (3.65–5.36)
		Moderate	3.09 ± 0.41	4.10 ± 0.36	t=12.07	<0.001	<0.001	d=2.66 (2.10–3.23)
		High	3.32 ± 0.45	3.95 ± 0.40	t=5.15	<0.001	<0.001	d=1.50 (0.87–2.12)
		Interaction P-value					<0.001	
	Metastasis Status	No metastasis	3.15 ± 0.43	4.20 ± 0.37	t=13.11	<0.001	<0.001	d=2.67 (2.14–3.19)
		Cervical lymph node	3.02 ± 0.41	4.08 ± 0.36	t=10.58	<0.001	<0.001	d=2.79 (2.09–3.49)
		Lung metastasis	2.90 ± 0.40	3.96 ± 0.39	t=7.18	<0.001	<0.001	d=2.69 (1.69–3.69)
		Cervical + Lung metastasis	2.82 ± 0.39	3.89 ± 0.41	t=5.21	<0.001	<0.001	d=2.66 (1.29–4.02)
		Interaction P-value					1.000	
Good treatment compliance	Age	13–18 years	16/32 (50.0%)	52/58 (89.7%)	χ²=17.56	<0.001	<0.001	OR=8.67 (2.91–25.85)
		19–25 years	31/53 (58.5%)	62/77 (80.5%)	χ²=7.48	0.006	0.018	OR=2.93 (1.34–6.43)
		Interaction P-value					0.115	
	Gender	Male	18/32 (56.2%)	43/54 (79.6%)	χ²=5.33	0.021	0.046	OR=3.04 (1.16–7.96)
		Female	29/53 (54.7%)	71/81 (87.7%)	χ²=18.36	<0.001	<0.001	OR=5.88 (2.50–13.81)
		Interaction P-value					0.316	
	Social Support	Low	13/29 (44.8%)	40/46 (87.0%)	χ²=15.23	<0.001	<0.001	OR=8.21 (2.66–25.34)
		Moderate	21/35 (60.0%)	50/59 (84.7%)	χ²=7.28	0.007	0.019	OR=3.70 (1.39–9.87)
		High	13/21 (61.9%)	24/30 (80.0%)	χ²=2.03	0.154	0.257	OR=2.46 (0.70–8.64)
		Interaction P-value					0.348	
	Metastasis Status	No metastasis	24/42 (57.1%)	60/68 (88.2%)	χ²=13.91	<0.001	<0.001	OR=5.62 (2.16–14.66)
		Cervical lymph node	14/25 (56.0%)	33/39 (84.6%)	χ²=6.39	0.011	0.029	OR=4.32 (1.33–13.99)
		Lung metastasis	6/12 (50.0%)	14/18 (77.8%)	χ²=2.50	0.114	0.202	OR=3.50 (0.72–17.09)
		Cervical + Lung metastasis	3/6 (50.0%)	7/10 (70.0%)	χ²=0.64	0.424	0.555	OR=2.33 (0.29–18.97)
		Interaction P-value					0.877	
Fertility confidence	Age	13–18 years	17/32 (53.1%)	48/58 (82.8%)	χ²=9.03	0.003	0.008	OR=4.24 (1.60–11.20)
		19–25 years	37/53 (69.8%)	55/77 (71.4%)	χ²=0.04	0.842	0.891	OR=1.08 (0.50–2.33)
		Interaction P-value					0.031	
	Gender	Male	20/32 (62.5%)	40/54 (74.1%)	χ²=1.28	0.259	0.374	OR=1.71 (0.67–4.39)
		Female	44/53 (83.0%)	63/81 (77.8%)	χ²=0.55	0.460	0.566	OR=0.72 (0.29–1.74)
		Interaction P-value					0.186	
	Social Support	Low	15/29 (51.7%)	36/46 (78.3%)	χ²=5.76	0.016	0.038	OR=3.36 (1.22–9.23)
		Moderate	27/35 (77.1%)	45/59 (76.3%)	χ²=0.01	0.923	0.958	OR=0.95 (0.35–2.57)
		High	12/21 (57.1%)	22/30 (73.3%)	χ²=1.46	0.227	0.347	OR=2.06 (0.63–6.74)
		Interaction P-value					0.213	
	Metastasis Status	No metastasis	32/42 (76.2%)	55/68 (80.9%)	χ²=0.35	0.557	0.651	OR=1.32 (0.52–3.36)
		Cervical lymph node	20/25 (80.0%)	30/39 (76.9%)	χ²=0.08	0.771	0.849	OR=0.83 (0.24–2.85)
		Lung metastasis	8/12 (66.7%)	12/18 (66.7%)	χ²=0.00	1.000	1.000	OR=1.00 (0.21–4.71)
		Cervical + Lung metastasis	4/6 (66.7%)	6/10 (60.0%)	χ²=0.07	0.790	0.852	OR=0.75 (0.09–6.23)
		Interaction P-value					0.925	
Overall satisfaction	Age	13–18 years	15/32 (46.9%)	50/58 (86.2%)	χ²=15.90	<0.001	<0.001	OR=7.08 (2.56–19.63)
		19–25 years	35/53 (66.0%)	48/77 (62.3%)	χ²=0.19	0.666	0.748	OR=0.85 (0.41–1.77)
		Interaction P-value					<0.001	
	Gender	Male	19/32 (59.4%)	38/54 (70.4%)	χ²=1.09	0.297	0.419	OR=1.62 (0.65–4.06)
		Female	31/53 (58.5%)	60/81 (74.1%)	χ²=3.57	0.059	0.116	OR=2.03 (0.97–4.24)
		Interaction P-value					0.712	
	Social Support	Low	12/29 (41.4%)	38/46 (82.6%)	χ²=13.61	<0.001	<0.001	OR=6.73 (2.33–19.46)
		Moderate	22/35 (62.9%)	42/59 (71.2%)	χ²=0.70	0.402	0.540	OR=1.46 (0.60–3.55)
		High	16/21 (76.2%)	18/30 (60.0%)	χ²=1.46	0.227	0.347	OR=0.47 (0.14–1.62)
		Interaction P-value					0.005	
	Metastasis Status	No metastasis	26/42 (61.9%)	52/68 (76.5%)	χ²=2.67	0.102	0.187	OR=2.00 (0.87–4.62)
		Cervical lymph node	16/25 (64.0%)	29/39 (74.4%)	χ²=0.78	0.376	0.517	OR=1.63 (0.55–4.84)
		Lung metastasis	5/12 (41.7%)	12/18 (66.7%)	χ²=1.83	0.176	0.284	OR=2.80 (0.62–12.66)
		Cervical + Lung metastasis	3/6 (50.0%)	5/10 (50.0%)	χ²=0.00	1.000	1.000	OR=1.00 (0.13–7.57)
		Interaction P-value					0.867	

Continuous data are presented as mean ± standard deviation and compared using independent-samples t-test; categorical data are presented as n (%) and compared using χ² test. FDR, false discovery rate (Benjamini-Hochberg procedure). OR, odds ratio; d, Cohen’s d. Interaction P-values were derived from Woolf’s test (categorical outcomes) or test of homogeneity of effect sizes (continuous outcomes). Subgroups with expected cell counts < 5 (e.g., cervical + lung metastasis) should be interpreted with caution; Fisher’s exact test is recommended for such small samples.For each subgroup, the interaction effect estimate (odds ratio or mean difference) and its 95% CI are provided.

Age subgroup: The intervention was associated with higher return-to-school/work rates in both age groups, but the effect was more pronounced in the 13–18 years subgroup (93.1% vs. 75.0%, FDR-P=0.037) than in the 19-25 years subgroup (88.3% vs. 81.1%, FDR-P=0.374); however, the interaction was not statistically significant (*P*_interaction =0.256). Treatment compliance improved significantly in both age groups (younger: 89.7% vs. 50.0%, FDR-P<0.001; older: 80.5% vs. 58.5%, FDR-P=0.018), with a non-significant interaction( P=0.115). Career planning clarity improved in both groups (both P<0.001), with a significant age interaction (P<0.001), suggesting a larger effect in younger patients. Fertility confidence improved significantly only in the younger subgroup (82.8% vs. 53.1%, FDR-P=0.008; interaction P = 0.031), while overall satisfaction also showed a significant interaction (P<0.001).

Gender subgroup: Female patients showed significant improvements in return-to-school/work (92.6% vs. 77.4%, FDR-P=0.029) and treatment compliance (87.7%vs. 54.7%, FDR-P<0.001), while male patients showed significant improvement only in treatment compliance (79.6% vs. 56.2%, FDR-P=0.046) and career planning clarity (both P<0.001). However, no gender interaction reached statistical significance for any outcome (all interaction P≥0.05). For fertility confidence, neither sex showed a significant intervention effect (female: 77.8% vs. 83.0%, P = 0.460; male: 74.1% vs. 62.5%, P = 0.251; interaction P = 0.34).

Social support subgroup: Patients with low social support experienced the largest improvements across most outcomes. For example, treatment compliance improved from 44.8% to 87.0% (FDR-P<0.001), overall satisfaction from 41.4% to 82.6% (FDR-P<0.001), and fertility confidence from 51.7% to 78.3% (FDR-P=0.038). The interaction for overall satisfaction was significant( P=0.005), indicating that the intervention effect on satisfaction was modified by social support level. Other interaction P values were non-significant (all >0.05).

Metastasis status subgroup: Patients without metastasis and those with isolated cervical lymph node metastasis showed significant improvements in treatment compliance (no metastasis: 88.2% vs. 57.1%, FDR-P<0.001; cervical: 84.6% vs. 56.0%, FDR-P=0.029), while patients with lung or combined metastasis did not show statistically significant improvements (all FDR-P>0.05). Career planning clarity improved significantly across all metastasis subgroups (all P<0.001), with no interaction P=1.000.

In summary, exploratory subgroup analyses revealed several numerical differences in intervention effects, particularly for younger patients and those with low social support. However, formal interaction tests were statistically significant only for a few outcomes (career planning clarity by age, fertility confidence by age, overall satisfaction by age and by social support). Given the exploratory nature and the limited number of significant interactions, these findings should be considered hypothesis-generating and require confirmation in future studies.

## 4 Discussion

This retrospective before-after cohort study examined the association between implementation of a systematic adolescent-centered supportive care program and outcomes in adolescents undergoing ^131^I therapy for DTC. After adjustment for potential confounders and sensitivity analyses using IPTW, program implementation was associated with significantly lower anxiety and depression scores, higher treatment compliance, improved social adaptation, and greater satisfaction. Exploratory subgroup analyses indicated suggestive differential intervention benefits according to age and social support level; nevertheless, the majority of interaction tests failed to reach statistical significance, such that these subgroup disparities remain hypothesis-generating and cannot be regarded as definitive evidence of effect modification.

Adolescence is characterized by the central developmental task of ego identity formation versus role confusion (43). AYA cancer, defined as cancer occurring in individuals aged 15-39,was analyzed using data from the Global Burden of Disease (GBD) 2021 study and the Global Cancer Observatory (GLOBOCAN) 2022 project (44). The growing burden of thyroid cancer among AYA populations demonstrates critical gender and geographic disparities, disproportionately affecting females and lower-resource regions (45). Radioactive iodine-131 therapy imposes physical isolation, radiation-related stigma, and uncertainty about long-term outcomes, disrupting identity construction (46). Systematic supportive care may facilitate the integration of illness experience into self-identity, resolves role confusion, and re-establishes developmental continuity.

Mitigation requires enhanced early detection protocols, optimized treatment pathways, and targeted resource allocation to vulnerable populations (47). Adolescents may demonstrate heightened sensitivity to psychosocial interventions compared with adults, which may explain the more pronounced associations observed in this study relative to some adult-focused research. Distinct from prior studies limited to psychological support, this model integrates career counseling, fertility guidance, social adaptation training, and family intervention to directly address AYA-specific developmental priorities.

Subgroup stratification was implemented to explore potential heterogeneity in intervention effectiveness. Although younger patients and those with low social support appeared to derive greater benefits from the intervention, statistically significant interaction effects were only scattered across a limited set of outcomes (48). Ma also found that gender, financial burden, personality trait, and need for adolescent-centered supportive care in nursing explained 24.5% of the total variance in the model and were independent predictors of psychological distress (49). These vulnerable subgroups may present with more severe baseline psychological distress and greater unmet developmental demands in social reintegration, career planning, and fertility expectation (50). While the majority of interaction comparisons failed to achieve statistical significance, and these stratified analyses were exploratory in nature without comprehensive correction for multiple testing, we refrain from drawing definitive conclusions regarding subgroup-specific efficacy. The age-related interaction for fertility confidence (P = 0.031) indicates that the intervention’s effect on this outcome may be more relevant for younger adolescents, who are at an earlier stage of reproductive life planning and may have greater unmet informational needs. Any apparent disparities across subgroups should primarily serve to generate hypotheses for future investigation, rather than directly guiding stratified clinical practice at present. Stratification by gender and metastasis status did not identify consistent modifiers of intervention responses.

Systematic supportive care was associated with robust and durable reductions in anxiety and depression symptoms. The control group demonstrated no spontaneous improvement in emotional distress, suggesting that active psychosocial intervention may be important for this cohort. Previous research has found that patients with psychiatric comorbidities and cancer were significantly less likely to benefit from screening, treatment, and end-of-life care, yet paradoxically were also less likely to be referred for psychosocial support (51). The program was also associated with significantly improved mastery of treatment-related knowledge, particularly future-oriented information concerning career development and reproductive health, which conforms to the knowledge-attitude-practice (KAP) theoretical model (52, 53). Elevated health literacy directly promoted compliance with isolation protocols, radioactive administration, and longitudinal follow-up. The intervention was associated with accelerated social reintegration,including return-to-school/return-to-work, social participation, and interpersonal relationship reconstruction (54). It was also associated with enhanced career planning clarity. Although fertility confidence did not improve significantly in the overall sample, the intervention showed a significant benefit in the younger age subgroup (13–18 years), suggesting that age-specific developmental concerns may influence the responsiveness to fertility-related counseling. These findings support whole-person recovery beyond oncological cure, while highlighting the need for tailored approaches to reproductive health education.

Active listening and empathic engagement may legitimize patients’ illness narratives, fear of radiation, and developmental concerns, reducing emotional suppression and psychological alienation. Personal narratives can be operationalized into targeted interventions to correct misconceptions about radiation, resolve identity crisis, and address unmet developmental needs. Therapeutic alliance, peer support networks, and family system strengthening may foster a sense of belonging and mitigate isolation-induced distress. Evidence-based education may correct cognitive distortions, alleviates negative affect, improves treatment adherence, promotes social and developmental adaptation (55). The intervention addresses biological (treatment safety, symptom management),psychological (anxiety, depression, self-stigma),and social (family function, peer relationship, social role) domains simultaneously, consistent with the holistic biopsychosocial model of health and illness.

These findings suggest that systematic supportive care programs may be a valuable component of routine radioactive iodine-131 therapy for adolescent thyroid cancer. Developmentally tailored educational protocols should extend beyond oncological and radiological parameters to address career trajectory, reproductive health, social interaction, and long-term quality of life. Personalized support may be prioritized for younger patients, female patients, and those with inadequate social support, although this requires further investigation. Formal collaboration with families, schools, and employers may be beneficial to streamline reintegration into academic and occupational settings. Peer mentorship programs should be considered to reduce stigma, enhance self-efficacy, and improve adaptive functioning.

There are still some limitations of this research. First, the retrospective before-after design using historical controls is vulnerable to secular trends, changes in clinical practice, staff experience, and healthcare system changes over time. Although we adjusted for measured confounders using multivariable regression and IPTW, residual confounding from unmeasured variables cannot be excluded. Second, the lack of assessor blinding for some outcomes may have introduced detection bias. We observed a slight baseline imbalance in occupation (SMD = 0.29) attributable to the historical control design, then we addressed this through comprehensive multivariable adjustment and IPTW sensitivity analyses. The consistent effect estimates across different analytical approaches suggest that our primary findings are unlikely to be driven by this specific covariate imbalance. Third, the complex, multimodal nature of the intervention precludes identification of which specific component(s) may be driving the observed associations. Fourth, the single-center design limits generalizability. Fifth, some outcome measures were based on self-designed or single-item instruments with limited prior validation in this population. Sixth, the relatively short follow-up (2 years) precludes assessment of very long-term developmental and reproductive outcomes. Seventh, although we applied FDR correction for multiple comparisons, the number of secondary outcomes increases the risk of Type I error; secondary and subgroup findings should be considered exploratory. Eighth, our analysis was based on a complete-case cohort after excluding 273 patients with incomplete data. This may introduce selection bias, and the distribution and reasons for exclusion may differ between the historical control and intervention periods, potentially affecting the generalizability of our findings.

## 5 Conclusion

In this retrospective before-after cohort study, implementation of a systematic adolescent-centered supportive care program was associated with reduced psychological distress, enhanced treatment adherence, and improved social reintegration. While overall fertility confidence did not show a statistically significant change, a significant improvement was observed in the younger age subgroup, warranting further investigation in adolescents undergoing ^131^I therapy for differentiated thyroid cancer. The findings support the feasibility and potential benefit of such programs in nuclear medicine practice. However, given the study’s observational design and inherent limitations, these results should be considered preliminary. Prospective, multicenter, randomized controlled trials are needed to establish causal efficacy and to identify which patient subgroups may benefit most from specific intervention components.

## Data Availability

The original contributions presented in the study are included in the article/**Supplementary Material**. Further inquiries can be directed to the corresponding author.

## Supplementary material

Supplementary Table 1 Linear Mixed-Effects Model Fit Statistics and Variance Components for SAS and SDS. Estimation method: Restricted Maximum Likelihood (REML); Covariance structure: Unstructured (selected based on lowest AIC/BIC among candidate structures: compound symmetry, first-order autoregressive, and unstructured);Fixed effects: Group (intervention vs. control), Time (categorical: baseline, 1m, 6m, 12m), Group×Time interaction, and baseline score of the respective outcome (SAS or SDS) as a covariate;Random effects: Random intercept for each participant;Degrees of freedom: Satterthwaite approximation.

## References

[1] Reverter JL . Thyroid cancer. Med Clin (Barc). (2025) 164:421–8. doi: 10.1016/j.medcli.2024.12.005 39880774

[2] Fisher SB Wang TS . Landmark studies in differentiated thyroid cancer. Ann Surg Oncol. (2025) 32:4729–41. doi: 10.1245/s10434-025-17419-1 40343589

[3] Sun Y Zhao Y Sun D Mu X Li J Lu C . Reflective analysis on the current (131)I adjuvant therapy indications in intermediate- and high-risk differentiated thyroid cancer. Eur J Nucl Med Mol Imaging. (2025) 52:3125–34. doi: 10.1007/s00259-025-07153-x 40055209

[4] Ma T Xie Y Long X Ye F . High risk factors, molecular features and clinical management for radioactive iodine-refractory differentiated thyroid carcinoma. Front Oncol. (2025) 15:1644562. doi: 10.3389/fonc.2025.1644562 40927522 PMC12414930

[5] Li M Dal Maso L Pizzato M Rumgay H Vaccarella S . Thyroid cancer in adolescents and young adults: a population-based study in 185 countries worldwide. Lancet Diabetes Endocrinol. (2026) 14:112–22. doi: 10.1016/s2213-8587(25)00289-x 41274302

[6] Qian J Qi S Hu R Zhang M Chen Z Ding Z . Trends in the disease burden of thyroid cancer among adolescents and young adults: a comparative study of China and global estimates (1990–2021). PloS One. (2025) 20:e0333373. doi: 10.1371/journal.pone.0333373 41086237 PMC12520391

[7] Goldfarb M Casillas J . Unmet information and support needs in newly diagnosed thyroid cancer: comparison of adolescents/young adults (AYA) and older patients. J Cancer Surviv. (2014) 8:394–401. doi: 10.1007/s11764-014-0345-7 24570216

[8] Massimino M Evans DB Podda M Spinelli C Collini P Pizzi N . Thyroid cancer in adolescents and young adults. Pediatr Blood Cancer. (2018) 65:e27025. doi: 10.1002/pbc.27025 29528191

[9] Portes PR Sandhu DS Longwell-Grice R . Understanding adolescent suicide: a psychosocial interpretation of developmental and contextual factors. Adolescence. (2002) 37:805–14. 12564830

[10] Santi D Spaggiari G Granata ARM Piticchio T Paoli D Lombardo F . Radioactive iodine and male reproductive health in thyroid cancer survivors: evidence of delayed gonadal dysfunction. J Endocrinol Invest. (2026) 49:299–308. doi: 10.1007/s40618-025-02725-y 41091412 PMC12924863

[11] Cai Y Yang Y Pang X Li S . The effect of radioactive iodine treatment for differentiated thyroid cancer on male gonadal function: a meta-analysis. Endocr Connect. (2023) 12. doi: 10.1530/ec-23-0299 37855387 PMC10692683

[12] Cui Y Jia Z Xie X . Nursing experience of adolescent patients with differentiated thyroid cancer treated by radioactive iodine‑131. J Basic Clin Oncol. (2022) 35(6):528–30.

[13] Lin CY Lin CL Kao CH . Risk of infertility in reproductive-age patients with thyroid cancer receiving or not receiving 131I treatment: a nationwide population-based cohort study. Clin Nucl Med. (2025) 50:201–7. doi: 10.1097/rlu.0000000000005570 39894986

[14] Puga FM Correia L Vieira I Caetano JS Cardoso R Dinis I . Differentiated thyroid cancer in children and adolescents: 12-year experience in a single center. J Clin Res Pediatr Endocrinol. (2024) 16:314–22. doi: 10.4274/jcrpe.galenos.2024.2024-1-25 38683018 PMC11590767

[15] Xiao Y Gu C Liu L Zeng X . Relationships among fertility concerns, fear of cancer recurrence, social support, self-efficacy, and family resilience among Chinese adolescents and young adults with cancer: a structural equation modeling. PloS One. (2026) 21:e0341351. doi: 10.1371/journal.pone.0341351 41628112 PMC12863571

[16] Charon R Ship A Asch SM . Arts, humanities, medicine, and discovery: a creative calling. J Gen Intern Med. (2020) 35:407–8. doi: 10.1007/s11606-019-05513-6 31792859 PMC7018877

[17] Xia C Wang B . Spatial and temporal trend analysis of the burden of endocrine-related cancers among women of reproductive age in the Asia-Pacific region from 1990 to 2021: results based on the GBD study. Front Oncol. (2025) 15:1678501. doi: 10.3389/fonc.2025.1678501 41647841 PMC12871539

[18] Volpe F Nappi C Zampella E Di Donna E Maurea S Cuocolo A . Current advances in radioactive iodine-refractory differentiated thyroid cancer. Curr Oncol. (2024) 31:3870–84. doi: 10.3390/curroncol31070286 39057158 PMC11276085

[19] Lin YS Wang RF Huang R Wen Q Cao W Chen LB . Chinese management guidelines for radioactive iodine-refractory differentiated thyroid cancer (2025 edition). Eur J Nucl Med Mol Imaging. (2025) 52:3859–76. doi: 10.1007/s00259-025-07222-1 40128355 PMC12316754

[20] Enomoto K Enomoto Y Uchino S Yamashita H Noguchi S . Follicular thyroid cancer in children and adolescents: clinicopathologic features, long-term survival, and risk factors for recurrence. Endocr J. (2013) 60:629–35. doi: 10.1507/endocrj.ej12-0372 23327804

[21] Eaglehouse YL Darmon S Gage MM Shriver CD Zhu K . Patient age at diagnosis and biological sex in association with postoperative outcomes of thyroidectomy for low-risk papillary thyroid cancer in the U.S. Military Health System. Gland Surg. (2025) 14:2187–99. doi: 10.21037/gs-2025-248 41377873 PMC12685778

[22] Huang W Zheng Y Guo J Luo C . The relationship between sleep disturbances, depression and anxiety in differentiated thyroid cancer patients during radioiodine (131I) therapy: a longitudinal observational study. Front Endocrinol (Lausanne). (2026) 17:1743676. doi: 10.3389/fendo.2026.1743676 41704488 PMC12907198

[23] Guo M Huang P . Impact of integrated nursing intervention combined with psychological care on postoperative quality of life, negative emotions, and nursing satisfaction in patients with thyroid cancer. Front Med (Lausanne). (2025) 12:1689245. doi: 10.3389/fmed.2025.1689245 41438138 PMC12719479

[24] Gowrishankar S Thompson D Bartz SK Johnstone LS Patel K Shah C . Pediatric thyroid cancer multidisciplinary team improves adherence of radioactive iodine treatment according to national guidelines. Otolaryngol Head Neck Surg. (2025) 173:1507–13. doi: 10.1002/ohn.1376 40778472 PMC12661476

[25] Ashkar C Sztal-Mazer S Topliss DJ . How to manage Graves' disease in women of childbearing potential. Clin Endocrinol (Oxf). (2023) 98:643–8. doi: 10.1111/cen.14705 35192205

[26] Anderson C Fitz V Deal A Getahun D Kwan ML Mersereau JE . Pregnancy attempts among adolescent and young adult cancer survivors. Fertil Steril. (2023) 119:475–83. doi: 10.1016/j.fertnstert.2022.12.024 36539058 PMC9993144

[27] Alexander EK Pearce EN Brent GA Brown RS Chen H Dosiou C . 2017 guidelines of the American Thyroid Association for the diagnosis and management of thyroid disease during pregnancy and the postpartum. Thyroid. (2017) 27:315–89. doi: 10.1089/thy.2016.0457 28056690

[28] Kitahara CM Slettebø Daltveit D Ekbom A Engeland A Gissler M Glimelius I . Maternal health, pregnancy and offspring factors, and maternal thyroid cancer risk: a Nordic population-based registry study. Am J Epidemiol. (2023) 192:70–83. doi: 10.1093/aje/kwac163 36130211 PMC10144719

[29] Kitahara CM Slettebø Daltveit D Ekbom A Engeland A Gissler M Glimelius I . Maternal health, in-utero, and perinatal exposures and risk of thyroid cancer in offspring: a Nordic population-based nested case-control study. Lancet Diabetes Endocrinol. (2021) 9:94–105. doi: 10.1016/s2213-8587(20)30399-5 33347809 PMC7875310

[30] Li JH Zhou Q . Psychological distress in thyroid cancer patients: influencing factors and intervention strategies. World J Clin Oncol. (2025) 16:106249. doi: 10.5306/wjco.v16.i7.106249 40741208 PMC12305095

[31] Yang C Feng X Li J Jiang Y Zhang H Gao Y . Clinical knowledge, perceptions, and communication confidence regarding the development of thyroid cancer --a cross-sectional study of clinical medical students in Chongqing, Southwest China. Risk Manag Healthc Policy. (2023) 16:2101–11. doi: 10.2147/rmhp.s424052 37849656 PMC10577241

[32] Hyun YG Alhashemi A Fazelzad R Goldberg AS Goldstein DP Sawka AM . A systematic review of unmet information and psychosocial support needs of adults diagnosed with thyroid cancer. Thyroid. (2016) 26:1239–50. doi: 10.1089/thy.2016.0039 27350421

[33] Haugen BR Alexander EK Bible KC Doherty GM Mandel SJ Nikiforov YE . 2015 American Thyroid Association management guidelines for adult patients with thyroid nodules and differentiated thyroid cancer: the American Thyroid Association Guidelines Task Force on Thyroid Nodules and Differentiated Thyroid Cancer. Thyroid. (2016) 26:1–133. doi: 10.1089/thy.2015.0020 26462967 PMC4739132

[34] Vollmar HC Ostermann T Redaèlli M . Using the scenario method in the context of health and health care--a scoping review. BMC Med Res Methodol. (2015) 15:89. doi: 10.1186/s12874-015-0083-1 26475601 PMC4609149

[35] Ward ZJ Gaba Q Atun R . Cancer incidence and survival for 11 cancers in the Commonwealth: a simulation-based modelling study. Lancet Oncol. (2024) 25:1127–34. doi: 10.1016/s1470-2045(24)00336-x 39127064

[36] Cetin N . Examining the role of post-treatment family support in pediatric and adolescent cancer survivors: a systematic review. J Adolesc Young Adult Oncol. (2023) 12:1–8. doi: 10.1089/jayao.2021.0179 35100040

[37] Neves MC Bártolo A Prins JB Sales CMD Monteiro S . Taking care of an adolescent and young adult cancer survivor: a systematic review of the impact of cancer on family caregivers. Int J Environ Res Public Health. (2023) 20. doi: 10.3390/ijerph20085488 37107768 PMC10138338

[38] Trask PC Paterson AG Trask CL Bares CB Birt J Maan C . Parent and adolescent adjustment to pediatric cancer: associations with coping, social support, and family function. J Pediatr Oncol Nurs. (2003) 20:36–47. doi: 10.1053/jpon.2003.5 12569433

[39] Cui P Shi J Li S Getu MA Wang R Chen C . Family resilience and its influencing factors among advanced cancer patients and their family caregivers: a multilevel modeling analysis. BMC Cancer. (2023) 23:623. doi: 10.1186/s12885-023-11101-z 37403053 PMC10320962

[40] Austin PC Stuart EA . Moving towards best practice when using inverse probability of treatment weighting (IPTW) using the propensity score to estimate causal treatment effects in observational studies. Stat Med. (2015) 34:3661–79. doi: 10.1002/sim.6607 26238958 PMC4626409

[41] Andrade C . Mean difference, standardized mean difference (SMD), and their use in meta-analysis: as simple as it gets. J Clin Psychiatry. (2020) 81. doi: 10.1093/jcpp.81 32965803

[42] Dralle H . 30 % Downstaging durch neue TNM-Stadieneinteilung der differenzierten Schilddrüsenkarzinome. Der Chirurg. (2019) 90:415. doi: 10.1007/s00104-019-0819-2 30778606

[43] Li W Liang H Wang W Liu J Liu X Lao S . Global cancer statistics for adolescents and young adults: population based study. J Hematol Oncol. (2024) 17:99. doi: 10.1186/s13045-024-01623-9 39434099 PMC11492650

[44] Danpanichkul P Pang Y Sirimangklanurak S Auttapracha T Pramotedham T Pan CW . Contemporary changes in global trends in early-onset cancer: incidence and mortality (2000-2021). Cancers (Basel). (2025) 17. doi: 10.3390/cancers17172766 40940863 PMC12427225

[45] Jia MJ Wang S Li Y Liu XN Jiang F Li HL . Global burden of thyroid cancer among adolescents and young adults, 1990-2021, and projections to 2050: an analysis based on the GBD 2021. Front Endocrinol (Lausanne). (2025) 16:1503144. doi: 10.3389/fendo.2025.1503144 40297175 PMC12034565

[46] LaFond CM Yost A Lankin K Kilaru M Cohn SL . The experience of children with neuroblastoma and their parents during single-room isolation for (131)I-metaiodobenzylguanidine therapy: a qualitative descriptive study. J Pediatr Hematol Oncol Nurs. (2022) 39:304–16. doi: 10.1177/27527530211068749 36129889

[47] Sun Y Wu J Tian H Qiu X Fang Y Xiao Y . Global research trends in palliative care for breast cancer from 2012 to 2022: a scientometric analysis. Front Oncol. (2023) 13:1104531. doi: 10.3389/fonc.2023.1104531 36910665 PMC9996305

[48] Wang S Wang X Liu X Zhao C Duan J . Moderating effects of humanistic care and socioeconomic status on the relationship among pain intensity, psychological factors, and psychological function in adults with cancer pain from a province of China: a cross-sectional study. Front Psychiatry. (2023) 14:928727. doi: 10.3389/fpsyt.2023.928727 37082761 PMC10110900

[49] Ma F Zhu Y Liu Y . The relationship between psychological distress and the nursing humanistic care demands in postoperative cancer inpatients: a cross-sectional study. BMC Nurs. (2024) 23:26. doi: 10.1186/s12912-024-01704-7 38195547 PMC10775573

[50] Wei J Dai D Sun Y Zheng W Gao M Liu Y . Empathy and humanistic care ability in oncology nurses: the mediating role of emotional intelligence. J Adv Nurs. (2025). doi: 10.1111/jan.70463 41408906 PMC13460961

[51] O'Connor J Bogue Kerr S . Cancer, care, and raining men: A call for humanity in the care of psychiatrized people. J Psychosoc Oncol. (2026), 1–11. doi: 10.1080/07347332.2026.2624025 41636539

[52] Ye X Xu T Cao J Li G Zhuang M Hu G . Knowledge, attitude, and practice toward genetic testing in breast cancer patients in China. PloS One. (2025) 20:e0322526. doi: 10.1371/journal.pone.0322526 40338860 PMC12061185

[53] Wu Y Zhang Z Qin W Chen W Xu T . Knowledge, attitudes, and practices regarding the postoperative management and TSH suppression therapy among patients with thyroid cancer. Front Oncol. (2025) 15:1441726. doi: 10.3389/fonc.2025.1441726 40134590 PMC11933125

[54] Ge H Qian Y Li B . Evaluation of knowledge, attitudes, and practices toward thyroid nodules in 456 patients with thyroid nodules. Med Sci Monit. (2025) 31:e945732. doi: 10.12659/msm.945732 39930692 PMC11829486

[55] Lehane E Leahy-Warren P O'Riordan C Savage E Drennan J O'Tuathaigh C . Evidence-based practice education for healthcare professions: An expert view. BMJ Evid Based Med. (2019) 24:103–8. doi: 10.1136/bmjebm-2018-111019 30442711 PMC6582731

