# Supplementary material for: Systematic adolescent-centered supportive care program is associated with improved psychological status, treatment adherence, and social adaptation in adolescents with thyroid cancer undergoing radioiodine-131 therapy: a retrospective before-after cohort study with historical controls
Source: Front Endocrinol (Lausanne). 2026 Sep 10;17:1901279. doi: 10.3389/fendo.2026.1901279 (PMC13600822; doi:10.3389/fendo.2026.1901279)
Supplement: Supplementary file 1 [file Supplementaryfile1.docx]

**Supplement1 Ethical Review Approval Letter（Translation version）**

Medical Ethics Committee of Northern Theater Command General Hospital

Ethical Review Approval Letter	Approval No.: YL(2021)07

| Item | Content |
| --- | --- |
| Full Study Title | Correlation Study of Systematic Humanistic Care on Psychological Status, Treatment Adherence and Social Adaptation in Adolescent Thyroid Cancer Patients Undergoing Radioiodine-131 Therapy |
| Project Source | Not Applicable |
| Research Grant No. | Not Applicable |
| Type of Clinical Research | Retrospective Observational Study |
| Principal Investigator | Zhang Guoxu |
| Department | Department of Nuclear Medicine |
| Venue of Review Meeting | Not Applicable |
| Date of Ethical Review | April 14, 2021 |
| Review Type | □ Full Committee Meeting Review ☑ Expedited Review □ Emergency Meeting Review |
| Submitted Documents | 1. Initial Review Application Form2. Research Protocol (Version 1.0, Dated April 2, 2021)3. Application for Waiver of Informed Consent4. Curriculum Vitae of Principal Investigator |
| Frequency of Annual / Periodic Continuing Review | Every 12 Months |

Important Provisions

If the investigator suspends or terminates the clinical research in advance, a suspension/termination report shall be submitted promptly.

A protocol deviation report shall be submitted if the research is conducted in violation of the approved protocol, or any circumstances that may adversely affect participants’ rights/health or the scientific validity of the study occur, which violate Good Clinical Practice (GCP) principles.

A written report shall be submitted without delay if any incident that may significantly interfere with the trial progress or increase risks to participants arises.

A final study report shall be submitted upon full completion of the clinical research.

This approval letter is valid for one year; it shall automatically expire if the research is not launched within the validity period.

Chairman’s Signature: ____________

Date: April 14, 2021
