# Supplementary material for: Systematic adolescent-centered supportive care program is associated with improved psychological status, treatment adherence, and social adaptation in adolescents with thyroid cancer undergoing radioiodine-131 therapy: a retrospective before-after cohort study with historical controls
Source: Front Endocrinol (Lausanne). 2026 Sep 10;17:1901279. doi: 10.3389/fendo.2026.1901279 (PMC13600822; doi:10.3389/fendo.2026.1901279)
Supplement: Supplementary file 2 [file Supplementaryfile2.docx]

**Supplement 2 The semi‑structured format core questionnaires**

The semi‑structured format core questionnaires

(1) “What does illness mean to you?”
(2) “What are you most worried about?”
(3) “What are your plans and expectations for the future?”
(4) “How do you think your family and friends see you?”
(5) “What do you hope to gain from this treatment?”
