# Supplementary material for: Systematic adolescent-centered supportive care program is associated with improved psychological status, treatment adherence, and social adaptation in adolescents with thyroid cancer undergoing radioiodine-131 therapy: a retrospective before-after cohort study with historical controls
Source: Front Endocrinol (Lausanne). 2026 Sep 10;17:1901279. doi: 10.3389/fendo.2026.1901279 (PMC13600822; doi:10.3389/fendo.2026.1901279)
Supplement: Supplementary file 3 [file Supplementaryfile3.docx]

**Supplement 3 Illustration related to radiation protection guidance for radioactive iodine-131 therapy**

Figure 1 Schematic diagram of physical properties and in vivo metabolic pathways of radioactive iodine-131:This figure illustrates the core physical properties of iodine-131 (half-life: 8.02 days, main radiation: short-range β-rays) and its in vivo metabolic process, including oral administration, selective uptake by the thyroid, and urinary excretion of unabsorbed fraction.

Figure 2: Radiation Level Attenuation Trend After Iodine-131 Therapy. The line chart quantifies the dynamic attenuation of relative radiation level in vivo after treatment, showing a significant decrease from 100% on treatment day to 2% at 1 month post-therapy.

Figure 3: Three Core Radiation Protection Measures for Home Isolation. This schematic presents three key protection strategies: distance protection (maintain 1-2m safe distance), time protection (minimize contact duration), and shielding protection (separate living arrangements).

Figure 4: Flow Chart for Returning to School/Work After Iodine-131 Therapy. The flow chart outlines the complete process for resuming normal life after treatment, including medical re-evaluation, unit reporting, collective protection, and regular follow-up.

Figure 5: Occupational Medical Examination Standards for Iodine-131 Treatment History. This table summarizes the entry medical examination requirements for special occupations with a history of radioactive iodine therapy, based on authoritative national policies.
