# Supplementary material for: Systematic adolescent-centered supportive care program is associated with improved psychological status, treatment adherence, and social adaptation in adolescents with thyroid cancer undergoing radioiodine-131 therapy: a retrospective before-after cohort study with historical controls
Source: Front Endocrinol (Lausanne). 2026 Sep 10;17:1901279. doi: 10.3389/fendo.2026.1901279 (PMC13600822; doi:10.3389/fendo.2026.1901279)
Supplement: Supplementary file 4 [file Supplementaryfile4.docx]

**Supplement 4: Career Planning Counseling Questionnaire**

**Instructions**: This questionnaire is designed to evaluate the effectiveness of career planning counseling for adolescent patients with thyroid cancer, as well as to understand patients’ needs and perceptions regarding career planning. Please read each question carefully and select the option that best matches your actual situation. All responses are anonymous and will only be used for academic research purposes.

**Rating Scale**: 1 = Strongly Disagree, 2 = Disagree, 3 = Agree, 4 = Strongly Agree

**Section 1: Basic Information (Optional)**

1. Gender: □ Male □ Female

2. Age: □ 12-15 years old □ 16-18 years old □ 19-22 years old

3. Educational level: □ Middle school □ High school □ College/University □ Other: _______

4. Whether you have a clear career direction: □ Yes □ No

5. If you have a clear career direction, please select the industry (multiple choices allowed): □ Civil aviation (pilot/attendant) □ Nuclear industry □ Food industry □ Other ordinary occupations □ Not sure

**Section 2: Career Direction Assessment**

1. The career planning counseling helped me clarify my career interests. □1 □2 □3 □4

2. The counseling process helped me recognize my own aptitudes and advantages related to my desired career. □1 □2 □3 □4

3. I have a clearer understanding of my career goals after the counseling. □1 □2 □3 □4

4. The counseling helped me assess whether my desired occupation (civil aviation/nuclear industry/food industry/other) is suitable for me. □1 □2 □3 □4

**Section 3: Career Limitation Clarification**

1. If your desired career is civil aviation: The counseling provided clear information on the entry medical examination standards for civil aviation personnel with a history of iodine-131 therapy. □1 □2 □3 □4

2. If your desired career is nuclear industry: The counseling helped me understand the occupational health requirements for nuclear industry workers with a history of iodine-131 therapy. □1 □2 □3 □4

3. If your desired career is food industry: The counseling clarified the health standards for food industry practitioners with my medical history. □1 □2 □3 □4

4. If you choose ordinary occupations: The counseling helped me understand whether my medical history will affect my employment in ordinary industries. □1 □2 □3 □4

5. I have a clear understanding of the impact of my medical history on my desired career choices after the counseling. □1 □2 □3 □4

**Section 4: Cognitive Restructuring**

1. The counseling helped me change the idea that “my medical history limits my career development”. □1 □2 □3 □4

2. The examples of successful people with illnesses shared in the counseling gave me confidence in pursuing my desired career (civil aviation/nuclear industry/food industry/other). □1 □2 □3 □4

3. I no longer feel “self-limited” due to my illness when considering my future career. □1 □2 □3 □4

4. I believe that I can still achieve my career goals despite my medical history. □1 □2 □3 □4

**Section 5: Success Story Sharing**

1. The experience sharing from former patients who successfully returned to work (especially those in my desired industry) was helpful to me. □1 □2 □3 □4

2. The success stories conveyed positive signals and enhanced my confidence in pursuing my desired career. □1 □2 □3 □4

3. I hope to have more opportunities to communicate with former patients who have successfully entered their desired occupations (civil aviation/nuclear industry/food industry/other). □1 □2 □3 □4

**Section 6: Academic Planning Support (For Student Patients)**

1. The counseling provided useful advice on the timing of suspension and resumption of school to better prepare for my desired career. □1 □2 □3 □4

2. I received effective guidance on how to connect my academic progress after returning to school, which is beneficial for my future career development. □1 □2 □3 □4

3. The counseling helped me master strategies for communicating with my school about my situation, to avoid potential impacts on my career planning. □1 □2 □3 □4

4. The counseling provided advice on academic improvement related to my desired career (e.g., professional courses for civil aviation/nuclear industry). □1 □2 □3 □4

**Section 7: Overall Evaluation and Suggestions**

1. Overall, I am satisfied with the career planning counseling. □1 □2 □3 □4

2. The career planning counseling met my needs for career development guidance (especially for my desired industry). □1 □2 □3 □4

3. Do you have any other suggestions for the career planning counseling, especially regarding the guidance for specific occupations?
