# Supplementary material for: Systematic adolescent-centered supportive care program is associated with improved psychological status, treatment adherence, and social adaptation in adolescents with thyroid cancer undergoing radioiodine-131 therapy: a retrospective before-after cohort study with historical controls
Source: Front Endocrinol (Lausanne). 2026 Sep 10;17:1901279. doi: 10.3389/fendo.2026.1901279 (PMC13600822; doi:10.3389/fendo.2026.1901279)
Supplement: Supplementary file 5 [file Supplementaryfile5.docx]

**Supplement 5: Follow-up Record Table for Adolescent Patients with Thyroid Cancer (Observation Group)**

| **Patient ID**  **(Anonymous)** | **Gender** | **Age at Treatment** | **Follow-up Time Point**  **(After Treatment)** | **Follow-up Content** | | | | | | | **Narrative Return Visit Records**  **(Key Content)** | **Follow-up Operator** | **Follow-up Date** |
| --- | --- | --- | --- | --- | --- | --- | --- | --- | --- | --- | --- | --- | --- |
|  |  |  |  | **Thyroid Function**  **(Normal/Abnormal/**  **Details)** | **Treatment Effect**  **(Excellent/Good/Fair/Poor)** | **Psychological Status**  **(Stable/Anxious/Depressed/Other)** | **Academic/**  **Career Progress**  **(Details)** | **Fertility Plan**  **(Has Plan/No Plan/Undecided/**  **Details)** | **Social Adaptation**  **(Good/General**  **/Poor/Details)** | **Other Relevant Conditions** |  |  |  |
|  | □ Male  □ Female |  | 1 month |  | □ Excellent  □ Good  □ Fair  □ Poor | □ Stable  □ Anxious  □ Depressed  □ Other: _______ |  | □ Has Plan  □ No Plan  □ Undecided  □ Details: _______ | □ Good  □ General  □ Poor  □ Details: _______ |  |  |  |  |
|  | □ Male  □ Female |  | 3 months |  | □ Excellent  □ Good  □ Fair  □ Poor | □ Stable  □ Anxious  □ Depressed  □ Other: _______ |  | □ Has Plan  □ No Plan  □ Undecided  □ Details: _______ | □ Good  □ General  □ Poor  □ Details: _______ |  |  |  |  |
|  | □ Male  □ Female |  | 6 months |  | □ Excellent  □ Good  □ Fair  □ Poor | □ Stable  □ Anxious  □ Depressed  □ Other: _______ |  | □ Has Plan  □ No Plan  □ Undecided  □ Details: _______ | □ Good  □ General  □ Poor  □ Details: _______ |  |  |  |  |
|  | □ Male  □ Female |  | 1 year |  | □ Excellent  □ Good  □ Fair  □ Poor | □ Stable  □ Anxious  □ Depressed  □ Other: _______ |  | □ Has Plan  □ No Plan  □ Undecided  □ Details: _______ | □ Good  □ General  □ Poor  □ Details: _______ |  |  |  |  |
|  | □ Male  □ Female |  | 2 years |  | □ Excellent  □ Good  □ Fair  □ Poor | □ Stable  □ Anxious  □ Depressed  □ Other: _______ |  | □ Has Plan  □ No Plan  □ Undecided  □ Details: _______ | □ Good  □ General  □ Poor  □ Details: _______ |  |  |  |  |

**Note**: This table is used to record the follow-up data of the observation group, which received more frequent follow-up. Follow-up time points: 1 month, 3 months, 6 months, 1 year, and 2 years after treatment. All records are kept confidential and only used for academic research.

**Explanation of Key Indicators**

1. Thyroid Function: Record specific indicators (e.g., TSH, FT3, FT4) if abnormal; mark “Normal” if all indicators are within the normal range.

2. Treatment Effect: Excellent = Complete remission, no recurrence; Good = Partial remission, no obvious complications; Fair = Stable condition, mild complications; Poor = Disease progression or severe complications.

3. Narrative Return Visit Records: Briefly record the patient’s description of the disease experience, life changes, and future plans (focus on key content of the narrative interview).

4. This table can be extended according to the number of patients; each patient corresponds to 5 follow-up time points (1 month, 3 months, 6 months, 1 year, 2 years).
