# Supplementary material for: Systematic adolescent-centered supportive care program is associated with improved psychological status, treatment adherence, and social adaptation in adolescents with thyroid cancer undergoing radioiodine-131 therapy: a retrospective before-after cohort study with historical controls
Source: Front Endocrinol (Lausanne). 2026 Sep 10;17:1901279. doi: 10.3389/fendo.2026.1901279 (PMC13600822; doi:10.3389/fendo.2026.1901279)
Supplement: Supplementary file 6 [file Supplementaryfile6.docx]

**Supplement 6: Self-Rating Anxiety Scale (SAS) and Self-Rating Depression Scale (SDS)**

Note: Both scales adopt 4-level scoring and are assessed at three time points: before treatment, 1 month after treatment, and 6 months after treatment. For SAS, a total score ≥50 indicates anxiety (Cronbach's α=0.86 in Chinese adolescents); for SDS, a total score ≥53 indicates depression (Cronbach's α=0.85 in Chinese adolescents).

**Table 1 Self-Rating Anxiety Scale (SAS)**

| **Item No.** | **Items** | **Scoring Standard**  **(4-level Scoring)** |
| --- | --- | --- |
| 1 | I feel nervous, anxious or on edge. | 1 = None or little of the time  2 = Some of the time  3 = Good part of the time  4 = Most or all of the time |
| 2 | I feel afraid as if something awful might happen. | 1 = None or little of the time  2 = Some of the time  3 = Good part of the time  4 = Most or all of the time |
| 3 | I feel restless and can't sit still. | 1 = None or little of the time  2 = Some of the time  3 = Good part of the time  4 = Most or all of the time |
| 4 | I feel that I might go crazy. | 1 = None or little of the time  2 = Some of the time  3 = Good part of the time  4 = Most or all of the time |
| 5 | I feel that I am a failure. | 1 = None or little of the time;  2 = Some of the time  3 = Good part of the time  4 = Most or all of the time |
| 6 | I feel downhearted and blue. | 1 = None or little of the time  2 = Some of the time  3 = Good part of the time  4 = Most or all of the time |
| 7 | I feel that others would be better off without me. | 1 = None or little of the time  2 = Some of the time  3 = Good part of the time  4 = Most or all of the time |
| 8 | I feel that I am not as good as others. | 1 = None or little of the time  2 = Some of the time  3 = Good part of the time  4 = Most or all of the time |
| 9 | I feel that I am worthless. | 1 = None or little of the time  2 = Some of the time  3 = Good part of the time  4 = Most or all of the time |
| 10 | I feel that I might hurt myself. | 1 = None or little of the time  2 = Some of the time  3 = Good part of the time  4 = Most or all of the time |
| 11 | I have trouble falling asleep or staying asleep. | 1 = None or little of the time  2 = Some of the time  3 = Good part of the time  4 = Most or all of the time |
| 12 | I feel tired and have no energy. | 1 = None or little of the time  2 = Some of the time  3 = Good part of the time  4 = Most or all of the time |
| 13 | I have trouble concentrating on things. | 1 = None or little of the time  2 = Some of the time  3 = Good part of the time  4 = Most or all of the time |
| 14 | I feel that I am slow in thinking. | 1 = None or little of the time  2 = Some of the time  3 = Good part of the time  4 = Most or all of the time |
| 15 | I feel that I am irritable. | 1 = None or little of the time  2 = Some of the time  3 = Good part of the time  4 = Most or all of the time |
| 16 | I have lost interest in things I used to enjoy. | 1 = None or little of the time  2 = Some of the time  3 = Good part of the time  4 = Most or all of the time |
| 17 | I have trouble making decisions. | 1 = None or little of the time; 2 = Some of the time; 3 = Good part of the time; 4 = Most or all of the time |
| 18 | I feel that my heart is racing. | 1 = None or little of the time 2 = Some of the time  3 = Good part of the time  4 = Most or all of the time |
| 19 | I feel short of breath. | 1 = None or little of the time 2 = Some of the time  3 = Good part of the time  4 = Most or all of the time |
| 20 | I feel that I have a dry mouth. | 1 = None or little of the time  2 = Some of the time  3 = Good part of the time  4 = Most or all of the time |

SAS Scoring Instructions: Sum the scores of all 20 items to get the total score. A total score ≥50 indicates anxiety. Cronbach's α=0.86 in Chinese adolescents.

**Table 2 Self-Rating Depression Scale (SDS)**

| **Item No.** | **Items** | **Scoring Standard**  **(4-level Scoring)** |
| --- | --- | --- |
| 1 | I feel down, depressed, or hopeless. | 1 = None or little of the time 2 = Some of the time  3 = Good part of the time  4 = Most or all of the time |
| 2 | I feel little interest or pleasure in doing things. | 1 = None or little of the time 2 = Some of the time  3 = Good part of the time  4 = Most or all of the time |
| 3 | I have trouble falling asleep, staying asleep, or sleeping too much. | 1 = None or little of the time 2 = Some of the time  3 = Good part of the time  4 = Most or all of the time |
| 4 | I feel tired or have little energy. | 1 = None or little of the time 2 = Some of the time  3 = Good part of the time  4 = Most or all of the time |
| 5 | I have a poor appetite or overeat. | 1 = None or little of the time  2 = Some of the time  3 = Good part of the time  4 = Most or all of the time |
| 6 | I feel bad about myself—or that I am a failure or have let myself or my family down. | 1 = None or little of the time 2 = Some of the time  3 = Good part of the time  4 = Most or all of the time |
| 7 | I have trouble concentrating on things, such as reading the newspaper or watching television. | 1 = None or little of the time 2 = Some of the time  3 = Good part of the time  4 = Most or all of the time |
| 8 | I move or speak so slowly that other people could have noticed. Or the opposite—I am so fidgety or restless that I have been moving around a lot more than usual. | 1 = None or little of the time  2 = Some of the time  3 = Good part of the time  4 = Most or all of the time |
| 9 | I have thoughts that I would be better off dead or of hurting myself in some way. | 1 = None or little of the time 2 = Some of the time  3 = Good part of the time  4 = Most or all of the time |
| 10 | I feel nervous, anxious or on edge. | 1 = None or little of the tim  2 = Some of the time  3 = Good part of the time  4 = Most or all of the time |
| 11 | I feel that I am irritable. | 1 = None or little of the time 2 = Some of the time  3 = Good part of the time  4 = Most or all of the time |
| 12 | I feel that I have no self-confidence. | 1 = None or little of the time  2 = Some of the time  3 = Good part of the time  4 = Most or all of the time |
| 13 | I feel that I am not as good as others. | 1 = None or little of the time 2 = Some of the time  3 = Good part of the time  4 = Most or all of the time |
| 14 | I have trouble making decisions. | 1 = None or little of the time 2 = Some of the time  3 = Good part of the time  4 = Most or all of the time |
| 15 | I feel that my heart is racing. | 1 = None or little of the time 2 = Some of the time  3 = Good part of the time  4 = Most or all of the time |
| 16 | I feel short of breath. | 1 = None or little of the time  2 = Some of the time  3 = Good part of the time  4 = Most or all of the time |
| 17 | I feel that I have a dry mouth. | 1 = None or little of the time 2 = Some of the time  3 = Good part of the time  4 = Most or all of the time |
| 18 | I feel that I am sweating a lot. | 1 = None or little of the time 2 = Some of the time  3 = Good part of the time  4 = Most or all of the time |
| 19 | I feel that I have trouble swallowing. | 1 = None or little of the time  2 = Some of the time  3 = Good part of the time  4 = Most or all of the time |
| 20 | I feel that I have a stomachache or discomfort. | 1 = None or little of the time 2 = Some of the time  3 = Good part of the time  4 = Most or all of the time |

SDS Scoring Instructions: Sum the scores of all 20 items to get the total score. A total score ≥53 indicates depression. Cronbach's α=0.85 in Chinese adolescents.
