# Supplementary material for: Systematic adolescent-centered supportive care program is associated with improved psychological status, treatment adherence, and social adaptation in adolescents with thyroid cancer undergoing radioiodine-131 therapy: a retrospective before-after cohort study with historical controls
Source: Front Endocrinol (Lausanne). 2026 Sep 10;17:1901279. doi: 10.3389/fendo.2026.1901279 (PMC13600822; doi:10.3389/fendo.2026.1901279)
Supplement: Supplementary file 7 [file Supplementaryfile7.docx]

**Supplement 7: Treatment-related Knowledge Mastery Questionnaire**

Note: This questionnaire is used to assess the mastery of treatment-related knowledge 1 month after treatment. The questionnaire was developed through literature review and expert panel review (5 experts including pediatric endocrinologists, nuclear medicine physicians, and psychologists), then pilot‑tested in 30 adolescent thyroid cancer patients prior to the study. There are 15 items in total, with a full score of 100 points. Each item is a single-choice question, scored as follows: A = 0 points (completely unknown), B = 3 points (basically unknown), C = 6 points (basically mastered), D = 10 points (proficiently mastered). The total score is the sum of scores of all items (range: 0–150 points), and converted to 100 points for evaluation. The evaluation criteria are as follows: ≥85 points = excellent, 60–84 points = qualified, <60 points = unqualified. Cronbach's α coefficient of this questionnaire is 0.89, and content validity index (CVI) is 0.92, indicating good reliability and validity.

**I. Treatment Principle (3 items)**

1. Do you understand the core principle of the current treatment (e.g., the mechanism of action, key treatment links)?
   A. Completely unknown B. Basically unknown C. Basically mastered D. Proficiently mastered
2. Do you know the main treatment objectives (e.g., disease control, symptom relief, quality of life improvement)?
   A. Completely unknown B. Basically unknown C. Basically mastered D. Proficiently mastered
3. Do you understand the possible treatment options and the reasons for choosing the current treatment plan?
   A. Completely unknown B. Basically unknown C. Basically mastered D. Proficiently mastered

**II. Radiation Protection (2 items)**

1. Do you know the correct methods of personal radiation protection during treatment (e.g., wearing protective equipment, keeping a safe distance)?
   A. Completely unknown B. Basically unknown C. Basically mastered D. Proficiently mastered
2. Do you understand the precautions for radiation protection of family members and surrounding people during your treatment period?
   A. Completely unknown B. Basically unknown C. Basically mastered D. Proficiently mastered

**III. Isolation Requirements (2 items)**

1. Do you know whether isolation is required during treatment and the specific isolation level (e.g., home isolation, hospital isolation)?
   A. Completely unknown B. Basically unknown C. Basically mastered D. Proficiently mastered
2. Do you understand the key points of isolation (e.g., isolation duration, daily living precautions, disinfection requirements)?
   A. Completely unknown B. Basically unknown C. Basically mastered D. Proficiently mastered

**IV. Side Effect Identification (2 items)**

1. Can you identify the common side effects of the current treatment (e.g., fatigue, nausea, skin reactions)?
   A. Completely unknown B. Basically unknown C. Basically mastered D. Proficiently mastered
2. Do you know the preliminary handling methods for mild side effects and the timing of seeking medical advice for severe side effects?
   A. Completely unknown B. Basically unknown C. Basically mastered D. Proficiently mastered

**V. Follow-up Schedule (2 items)**

1. Do you remember the specific time points of follow-up after treatment (e.g., 1 month, 3 months, 6 months after treatment)?
   A. Completely unknown B. Basically unknown C. Basically mastered D. Proficiently mastered
2. Do you know the main items of each follow-up (e.g., physical examination, laboratory tests, imaging examinations)?
   A. Completely unknown B. Basically unknown C. Basically mastered D. Proficiently mastered

**VI. Occupational Impact (2 items)**

1. Do you understand the possible impact of the current treatment on your future work (e.g., work intensity, work type)?
   A. Completely unknown B. Basically unknown C. Basically mastered D. Proficiently mastered
2. Do you know the relevant precautions and adaptation methods when returning to work after treatment?
   A. Completely unknown B. Basically unknown C. Basically mastered D. Proficiently mastered

**VII. Fertility Impact (2 items)**

1. Do you understand the possible impact of the current treatment on your fertility (male/female)?
   A. Completely unknown B. Basically unknown C. Basically mastered D. Proficiently mastered
2. Do you know the relevant protective measures and follow-up suggestions for fertility after treatment (if applicable)?
   A. Completely unknown B. Basically unknown C. Basically mastered D. Proficiently mastered

Scoring Method: Total raw score = sum of scores of 15 items (range: 0–150 points); Converted total score (100 points) = (raw score / 150) × 100. Evaluation Criteria: Converted total score ≥85 points = excellent; 60–84 points = qualified; <60 points = unqualified.
