# Supplementary material for: Systematic adolescent-centered supportive care program is associated with improved psychological status, treatment adherence, and social adaptation in adolescents with thyroid cancer undergoing radioiodine-131 therapy: a retrospective before-after cohort study with historical controls
Source: Front Endocrinol (Lausanne). 2026 Sep 10;17:1901279. doi: 10.3389/fendo.2026.1901279 (PMC13600822; doi:10.3389/fendo.2026.1901279)
Supplement: Supplementary file 8 [file Supplementaryfile8.docx]

**Supplement 8 Comprehensive Assessment of Related Indicators for Adolescent Patients with Thyroid Cancer Undergoing ¹³¹I Therapy**

**Assessment Time**: 6 months after ¹³¹I treatment

**General Note**: All assessment items are conducted 6 months after ¹³¹I treatment;

5-point scale is used for relevant items (1 = lowest/extremely low; 5 = highest/extremely high).

| **No.** | **Assessment Dimension** | **Assessment Items** | **Evaluation**  **(√ as applicable)** | **Assessment Criterion/Note** |
| --- | --- | --- | --- | --- |
| 1 | Treatment Compliance | Timely iodine-131 intake | □ Yes □ No | Good compliance = all items answered "Yes"; Poor compliance = one or more items answered "No"; Assessed by attending physicians. |
|  |  | Compliance with isolation requirements | □ Yes □ No |  |
|  |  | Timely follow-up | □ Yes □ No |  |
|  |  | Final Compliance Outcome | □ Good compliance  □ Poor compliance |  |
| 2 | Social Adaptation (Self-designed questionnaire) | Return-to-school/return-to-work status | □ Returned normally  □ Not returned  □ Partially returned (specify: _________) | 5-point scale for social participation and relationship satisfaction. |
|  |  | Social participation (degree of participation in social activities, gatherings, etc.) | □ 1 □ 2 □ 3 □ 4 □ 5 |  |
|  |  | Relationship satisfaction (satisfaction with family, friends and other interpersonal relationships) | □ 1 □ 2 □ 3 □ 4 □ 5 |  |
| 3 | Career Planning Clarity (Single-item questionnaire) | I have a clear plan for future career development | □ 1 (Extremely unclear)  □ 2 (Unclear)  □ 3 (Moderate)  □ 4 (Clear)  □ 5 (Extremely clear) | 5-point scoring. |
| 4 | Fertility Cognition and Confidence (Self-designed questionnaire) | Fertility knowledge mastery (5 items in total) | □ Excellent (≥80 points)  □ Non-excellent (<80 points) | Fertility confidence is evaluated by 5-point scale. |
|  |  | Fertility confidence | □ 1 □ 2 □ 3 □ 4 □ 5 |  |
| 5 | Satisfaction (Self-designed questionnaire) | Doctor-patient communication | □ 1 □ 2 □ 3 □ 4 □ 5 | Overall satisfaction = proportion of "very satisfied" (5 points) or "satisfied" (4 points). |
|  |  | Attention to psychological concerns | □ 1 □ 2 □ 3 □ 4 □ 5 |  |
|  |  | Usefulness of information support | □ 1 □ 2 □ 3 □ 4 □ 5 |  |
|  |  | Overall experience | □ 1 □ 2 □ 3 □ 4 □ 5 |  |
|  |  | Overall Satisfaction Outcome | □ Very satisfied  □ Satisfied  □ Moderate  □ Dissatisfied  □ Very dissatisfied |  |
| - | **Assessor (Attending Physician/Investigator) Signature** | | ________________________ | |
| - | **Assessment Date** | | ______ / ______ / ______ | |
