# Supplementary material for: Systematic adolescent-centered supportive care program is associated with improved psychological status, treatment adherence, and social adaptation in adolescents with thyroid cancer undergoing radioiodine-131 therapy: a retrospective before-after cohort study with historical controls
Source: Front Endocrinol (Lausanne). 2026 Sep 10;17:1901279. doi: 10.3389/fendo.2026.1901279 (PMC13600822; doi:10.3389/fendo.2026.1901279)
Supplement: Supplementary file 9 [file Table1.docx]

Supplementary Table S1. Linear Mixed-Effects Model Fit Statistics and Variance Components for SAS and SDS

| Outcome | Effect / Parameter | Estimate | 95% CI | df | P value | ‑2 Log Likelihood | AIC | BIC |
| --- | --- | --- | --- | --- | --- | --- | --- | --- |
| SAS | Group×Time interaction F | F= 18.42 | — | (3, 654) | <0.001 | 4826.35 | 4846.35 | 4884.12 |
|  | Adjusted mean difference at 6m (Intervention - Control) | -4.60 | (-6.12, -3.08) | 654 | <0.001 |  |  |  |
|  | Adjusted mean difference at 12m (Intervention - Control) | -7.40 | (-8.92, -5.88) | 654 | <0.001 |  |  |  |
|  | Random intercept variance (τ₀²) | 18.240 | (14.86, 22.38) |  |  |  |  |  |
|  | Residual variance (σ²) | 14.56 | (12.91, 16.45) |  |  |  |  |  |
|  | Intraclass correlation (ICC) | 0.556 |  |  |  |  |  |  |
| SDS | Group×Time interaction F | F = 22.56 | — | (3, 654) | <0.001 | 4918.72 | 4938.72 | 4976.49 |
|  | Adjusted mean difference at 6m (Intervention - Control) | -4.23 | (-5.94, -3.17) | 654 | <0.001 |  |  |  |
|  | Adjusted mean difference at 12m (Intervention - Control) | -4.25 | (-6.13, -2.95) | 654 | <0.001 |  |  |  |
|  | Random intercept variance (τ₀²) | 15.87 | (12.93, 19.48) |  |  |  |  |  |
|  | Residual variance (σ²) | 18.34 | (16.26, 20.71) |  |  |  |  |  |
|  | Intraclass correlation (ICC) | 0.464 |  |  |  |  |  |  |

*Notes:Estimation method: Restricted Maximum Likelihood (REML); Covariance structure: Unstructured (selected based on lowest AIC/BIC among candidate structures: compound symmetry, first‑order autoregressive, and unstructured);Fixed effects: Group (intervention vs. control), Time (categorical: baseline, 1m, 6m, 12m), Group×Time interaction, and baseline score of the respective outcome (SAS or SDS) as a covariate;Random effects: Random intercept for each participant;Degrees of freedom: Satterthwaite approximation*
